# ﻿A nomenclator of *Drymaria* (Caryophyllaceae)

**DOI:** 10.3897/phytokeys.266.162517

**Published:** 2025-11-13

**Authors:** Dagoberto Valentín-Martínez, Victor W. Steinmann, Hilda Flores-Olvera, Thomas Borsch, Daniel B. Montesinos-Tubée

**Affiliations:** 1 Instituto de Ecología, A.C., Centro Regional del Bajío, Av. Lázaro Cárdenas 253, 61600 Pátzcuaro, Michoacán, Mexico Instituto de Ecología Pátzcuaro Mexico; 2 Departamento de Botánica, Instituto de Biología, Universidad Nacional Autónoma de México, Ciudad de México, Mexico Universidad Nacional Autónoma de México Ciudad de México Mexico; 3 Botanischer Garten und Botanisches Museum Berlin, Freie Universität Berlin, Königin-Luise-Straße 6-8, 14195 Berlin, Germany Freie Universität Berlin Berlin Germany; 4 Universidad Científica del Sur, Ciencias de la Ingeniería, Carrera de Agronomía y Negocios, Campus Villa. Panamericana Sur Km 19, Villa, Lima, Peru Universidad Científica del Sur Lima Peru

**Keywords:** Caryophyllales, lectotype, neotype, nomenclature, Paronychioideae, Polycarpeae, species list

## Abstract

*Drymaria* (Caryophyllaceae) is here considered to contain 56 species plus 19 infraspecific taxa, excluding autonyms. In the current study, all effectively published names of *Drymaria* were compiled, and information about their taxonomy, distribution, and nomenclatural types is provided. In addition, an overview of the taxonomic history of *Drymaria* is presented. As a result, one neotype, 15 lectotypes, and the type for the genus *Mollugophytum* are newly designated. Altogether, 188 names were treated, of which 52 are heterotypic and 50 are homotypic synonyms. An additional 10 names were either unresolved or are now considered to belong to other genera. A brief assessment of the distribution of the respective taxa is also provided, including the examination of herbarium specimens and specimen images, which indicates that two regions possess high levels of endemism—Mexico with 20 species and the Andes with 14 species. There are few studies that assess the conservation status of *Drymaria* species, and only 10 species and two varieties have had IUCN Red List assessments.

## ﻿Introduction

The genus *Drymaria* Willd. belongs to the family Caryophyllaceae, tribe Polycarpeae (Greenberg and Donoghue 2011). It is a primarily American taxon with species diversity concentrated in subtropical regions from the western United States and Mexico to northern Argentina and Chile, and also in the Caribbean islands. Only three taxa occur in the Old World, one of which is introduced and two of which are considered native. Estimates of diversity in *Drymaria* have ranged from 48 to 57 species depending on the author (Hartman 2005; Pérez-Calix and Grajales-Tam 2013; Rodríguez-Jiménez 2013; Hernández-Ledesma et al. 2015; Arya et al. 2024). Two geographical regions possess high numbers of endemics: Mexico with 20 species and the Andes of Bolivia, Chile, Ecuador, and Peru with 14.

The etymological origin of *Drymaria* comes from the Greek word *drymos* and may refer to “wood,” in allusion to the oak forests where some of the species grow, or to “knotty,” in resemblance to the adventitious roots that often emerge from the nodes, giving the appearance of small forests (Muñoz-Schick et al. 2012).

Our current state of knowledge of *Drymaria* is based on morphological characters (e.g., Duke 1961), and comprehensive studies to clarify phylogenetic relationships within *Drymaria* or to test species limits with an evolutionary approach do not yet exist. Previous phylogenetic research has included only a few species in studies focused on resolving relationships within the Caryophyllales (Downie et al. 1997; Cuénoud et al. 2002; Brockington et al. 2009) or among major lineages of Caryophyllaceae (Fior et al. 2006; Harbaugh et al. 2010; Greenberg and Donoghue 2011). Greenberg and Donoghue (2011) and Zanotti et al. (2022) included four species of *Drymaria* in their research. However, in both cases this was not sufficient to demonstrate the monophyly of the genus, neither in terms of taxon sampling nor tree resolution.

Most of the species of *Drymaria* were described in the 19^th^ and early 20^th^ centuries, and only three species were proposed as new in the last 25 years: *D. jenniferae* (Villarreal and Estrada 2008), *D. veliziae* (Montesinos-Tubée et al. 2020), and *D. anilii* (Arya et al. 2024). By far the most important taxonomic treatment of *Drymaria* is Duke’s (1961) revision. Before that, contributions mostly entailed the description of new species or floristic treatments for specific geographic areas, and later taxonomic treatments were produced for regional floras, largely relying on Duke’s taxonomic concepts but without using any detailed research such as phylogeny or an integrative taxonomy approach.

The Caryophyllaceae and the genus *Drymaria* belong to the Caryophyllales, for which a research network was established 10 years ago (Borsch et al. 2015; Flores-Olvera et al. 2018; The Caryophyllales Network, https://caryophyllales.org/ [continuously updated]) that later became the Taxonomic Expert Network (TEN) for the order in World Flora Online. As an international collaborative initiative, the CaryophyllalesTEN has supported research towards a better understanding of the diversity of species of this group of plants and has synthesized taxonomic knowledge using electronic tools. For *Drymaria*, our goal was to elaborate an expertly revised species list as the first step of a more comprehensive ongoing analysis, more than 60 years after the last revision of the genus. Specifically, we aimed to compile all published names of *Drymaria* and revisit nomenclature and typification in order to ensure the correct application of all names and update the overall taxonomic treatment. This study is therefore intended to serve as a baseline reference for future floristic, phylogenetic, and monographic research on *Drymaria*.

## ﻿Methods

The basis of the list was Duke’s (1961) revision. Unless there was subsequent evidence to the contrary, Duke’s circumscription of taxa was followed, and both varieties and subspecies are recognized. Species described subsequently by Turner (1995), Villarreal and Estrada (2008), Montesinos-Tubée et al. (2020), and Arya et al. (2024) were added. To ensure that all names assigned to *Drymaria* were included, the online resources IPNI (International Plant Names Index, http://www.ipni.org), Tropicos (Tropicos, http://www.tropicos.org), POWO (Plants of the World Online, https://powo.science.kew.org/), and the latest version of the WFO (The World Flora Online, https://www.worldfloraonline.org/) were consulted. The taxonomic backbone for *Drymaria* in that version was preliminary, awaiting revision by specialists from the CaryophyllalesTEN.

Specimens from the following herbaria were examined: AC, ANSM, ARIZ, B, BM, C, CAS, CIIDIR, COLO, CPUN, DS, DUKE, E, F, FI, FT, G, GBH, G-DC, GH, GOET, HAL, ILL, JE, K, KFTA, L, LD, LE, LECB, LL, MEL, MEXU, MICH, MIN, MO, MOL, MSC, ND, NY, OS, P, PH, POM, PR, PUL, RM, RSA, S, SGO, TEX, UC, UNM, US, USM, VT, W, WIS, and YU (abbreviations according to Thiers 2025). When present on the specimen, the accession number and/or barcode number are provided after the citation of the herbarium acronym. When two numbers are given separated by a diagonal, for example “[1807078/144088],” the first number represents the accession number and the second number the barcode number. An exclamation point is used when the specimen was seen, either physically or as a photo. The following online resources were consulted to view specimen images: JSTOR Global Plants (ITHAKA 2024), Harvard University Herbaria & Libraries (https://kiki.huh.harvard.edu/databases/image_search.php), JACQ consortium (https://www.jacq.org/), Smithsonian National Museum of Natural History (https://collections.nmnh.si.edu/search/botany/), and Tropicos (Tropicos, https://tropicos.org/image/). In some instances, specimen images were provided directly by the curators of the herbaria BM, C, CPUN, F, G, L, PR, and US, because the specimens were not available online or because there were doubts about the existence of the specimens in the collections. Specimen information was compared with the protologues of all the taxa, and “Taxonomic Literature-2” (Stafleu and Cowan 1976, 1979, 1981, 1983, 1986, 1988) was frequently consulted for information about publication dates and other pertinent bibliographic information.

The distributions of the species were obtained from many sources, the most important of which were the GBIF (Global Biodiversity Information Facility, 2025), iNaturalist (iNaturalist, https://www.inaturalist.org/), POWO (Plants of the World Online, https://powo.science.kew.org/), and the protologues, as well as Duke’s (1961) revision and some floristic treatments, e.g., Macbride (1937), Standley and Steyermark (1946), Hartman (2005), Cano and Sánchez (2006), and Rodríguez-Jiménez (2013). In the case of taxa from Central and North America, the herbaria IEB, MEXU, POM, and RSA were examined. Specimens available online via SEINet (https://swbiodiversity.org/seinet/index.php) were also considered. For the Americas, all countries are given in the distribution assessment, but for taxa in the Old World, only the continent is listed. The lectotype and neotype designations were made following the rules provided in the current International Code of Nomenclature for Algae, Fungi, and Plants (Turland et al. 2018). For conservation status, local IUCN Red List books were checked (Cano and Sánchez 2006; León Yáñez et al. 2011). Preliminary evaluations included in the protologues of some recently described species (Montesinos-Tubée et al. 2020; Arya et al. 2024) are also given.

## ﻿Results

In total, 188 names are reported, 146 corresponding to the rank of species. Of these, we accept 56 species, five subspecies belonging to four different species, and 15 varieties belonging to eight of these species, not including autonyms. There are 52 heterotypic synonyms and 50 homotypic synonyms. Ten names are excluded. Two correspond to species currently accepted in *Stellaria* and one in *Odontostemma*. For the other seven, it is not possible to determine if they are members of *Drymaria* because of the lack of at least one specimen and/or a detailed description. One neotype, 15 lectotypes, and the type for the genus *Mollugophytum* are designated.

## ﻿Discussion

### ﻿History of the classification of *Drymaria*

The first species of the genus was described by Linnaeus (1753) as *Holosteum cordatum* L. Thirty-five years later, Swartz (1788) described a second taxon, *H. diandrum* Sw., but this is currently treated as a synonym of *D. cordata*. When Willdenow in Roemer and Schultes (1819) proposed the genus *Drymaria*, he transferred *H. cordatum* into it and described three additional taxa: *D. stellarioides* Willd., *D. ovata* Willd., and *D. arenarioides* Willd. Later, Britton and Millspaugh (1920) designated *D. arenarioides* as the type of the genus. However, these authors followed the largely mechanical method of choosing the first species as the type (see Art. 10.7 of the International Code; Turland et al. 2018), and their choice is superseded by Fosberg’s (1945) designation of *D. cordata* as the type. Four years after its description, Kunth (1823) proposed a fifth species of the genus, *D. divaricata*. At the time the genus was treated for de Candolle’s *Prodromus* (Seringe 1824), a total of five species were included. Since the 1820s, the number of species described has grown considerably, totaling more than 120 validly published names. Although a number of species of *Drymaria* were at some time erroneously included in *Alsine* L., *Arenaria* L., *Lepigonum* (Fr.) Wahlberg, *Polycarpon* Loefl., *Spergula* L., *Spergularia* (Pers.) J.Presl & C.Presl, or *Stellaria* L., the majority have always been included within *Drymaria*.

Four further species were described in “Reliquiae Haenkeanae” (Bartling 1831), three by Friedrich G. Bartling and one by Carl B. Presl: *D. apetala* Bartl., *D. glaberrima* Bartl., *D. glandulosa* C.Presl, and *D. grandiflora* Bartl. From 1849 to 1882, Asa Gray described a number of new taxa (Gray 1854): *D. effusa* A.Gray, *D. fasciculata* A.Gray, *D. polycarpoides* A.Gray, *D. rotundifolia* A.Gray, *D. suffruticosa* A.Gray, *D. xerophylla* A.Gray, and *D. viscidula* A.Gray, the latter of which is currently accepted as D. divaricata var. viscidula (A.Gray) J.A.Duke.

Two small segregate genera of *Drymaria* have been proposed: *Pinosia* Urb. (Urban 1930) and *Mollugophytum* M.E.Jones (Jones 1933), but neither of these gained general acceptance. The former was distinguished from *Drymaria* by having three petals, a free ovary, and above all by having subglobose, smooth, and 3-sulcate pollen grains. It included only *Drymaria ortegioides* Griseb. when first described. Later, Liogier (1960) proposed a second species, *Pinosia glandulosa* Alain. However, two years later (1962), Liogier himself considered the genus a synonym of *Drymaria*. *Mollugophytum* was initially circumscribed to include *D. crassifolia* Benth., *D. holosteoides* Benth., *D. polycarpoides*, and *D. sperguloides* A.Gray. Jones (1933) mentioned that this assemblage of species had no close relationship with *Drymaria* and characterized it by: 1) oval sepals lacking apiculation and nerves but with conspicuous and hyaline margins, 2) capsule equaling the sepals, oval-ovoid, 3–4 valved, smooth, shiny, and finely striate longitudinally with beaded lines, and 3) seeds 8, large, circinate, and longitudinally striate with low tubercles. However, shortly afterwards, Fosberg (1945) merged *Mollugophytum* with *Drymaria* and provided two new varietal combinations: D. glandulosa var. fendleri (S.Watson) Fosberg and var. perennis (M.E.Jones) Fosberg.

The first detailed regional floristic contribution was conducted by Macbride (1937) for Peru. He recognized 22 species and, together with Charles Baehni, described four species and proposed a new combination: *D. agapatensis* Baehni & J.F.Macbr., *D. devia* Baehni & J.F.Macbr., *D. praecox* Baehni & J.F.Macbr., *D. sphagnophylla* Baehni & J.F.Macbr., and *D. engleriana* (Muschl.) Baehni & J.F.Macbr. Wiggins (1944) treated the 15 species of the Sonoran Desert and adjacent areas. Although he described a new species, *D. johnstonii* Wiggins, and a new variety, D. carinata var. perennis Wiggins, these are currently considered synonyms of D. arenarioides subsp. peninsularis (S.F.Blake) J.A.Duke and D. gracilis subsp. carinata (Brandegee) J.A.Duke, respectively. Standley and Steyermark (1946) revised 10 species for the “Flora of Guatemala” as the first treatment of the genus for Central America.

Mizushima (1957) studied the morphological variation in the widespread *D. cordata* and concluded that the *D. cordata* complex comprises *D. diandra* Blume from Asia, Africa, and Oceania, and in the New World, *D. cordata* with two varieties: var. cordata in continental North and South America, and var. pacifica M.Mizush., endemic to the Galapagos Islands. Johnston (1940, 1950) revised the genus in the northern Chihuahuan Desert state of Coahuila and proposed three new species, *D. elata* I.M.Johnst., *D. lyropetala* I.M.Johnst., *D. subumbellata* I.M.Johnst., and the variety D. lyropetala var. coahuilana I.M.Johnst. Within the last 20 years, *Drymaria* has been treated in various regional floras, such as: “Flora Fanerogámica del Valle de México” (Calderón de Rzedowski and Rzedowski 2005), “Flora de Guerrero” (Castro-Mendoza and Fonseca 2012), “Flora del Bajío y de Regiones Adyacentes” (Pérez-Calix and Grajales-Tam 2013), “Flora del Valle de Tehuacán-Cuicatlán” (Machuca-Machuca 2024), “La familia Caryophyllaceae en el estado de Aguascalientes, México” (Sandoval-Ortega et al. 2019), “Flora of North America” (Hartman 2005), and “Flora Mesoamericana” (Rodríguez-Jiménez 2013).

The only detailed revision of the whole genus was made by Duke (1961). It was based on the morphological examination of herbarium specimens, including the types of most names. He recognized 48 species, two of which were described as new, and proposed 22 new varieties or new combinations for varieties. Duke described many infraspecific taxa and also made changes in status, treating taxa formerly published at the species rank at the level of variety. At this point, we have not tested these proposals, as this will require a phylogenetic approach and the use of DNA sequence data. We therefore maintain infraspecific taxa accepted by Duke (1961) for now and comment on their taxonomic status rather than sinking them into synonyms without convincing additional data.

Duke’s (1961) work also marks the first attempt at an infrageneric classification of the genus, which consisted of 17 series (see below). These were distinguished primarily based on floral characters. However, these series were not validly published because he did not include a Latin description or diagnosis (see Turland et al. 2018, Art. 39.1). Also, the series with more than one species had none of these designated as type. Although he proposed the series Cordatae, this should have been named ser. Drymaria since it contains the type of the genus, *D. cordata* (see Turland et al. 2018, Art. 22.1). Table 1 contains the 17 series proposed by Duke (1961) and includes the species subsequently described by Turner (1995), Villarreal and Estrada (2008), Montesinos-Tubée et al. (2020), and Arya et al. (2024). It is important to mention that Escamilla and Sosa (2000) used these series as a basis for their sampling for the study of seed morphology in *Drymaria*.

**Table 1.** “Series” proposed by Duke (1961) and the species included in each one, as well as the species described by Turner (1995), Villarreal and Estrada (2008), Montesinos-Tubée et al. (2020), and Arya et al. (2024).

Series	Species
* Arenarioides *	*D. arenarioides*, *D. axillaris*, *D. barkleyi*, *D. jenniferae*, *D. molluginea*, *D. pattersonii, D. polycarpoides*
* Cordatae *	*D. anilii*, *D. cordata*, *D. gracilis*, *D. glandulosa*, *D. ladewii*, *D. xerophylla*
* Debiles *	*D. debilis*
* Divaricatae *	*D. divaricata*
* Excisae *	*D. excisa*, *D. hypericifolia*, *D. longepedunculata*
* Fasciculatae *	*D. engleriana*, *D. fasciculata, D. praecox*
* Frutescentes *	*D. auriculipetala*, *D. frutescens*, *D. stellarioides*, *D. stereophylla, D. veliziae*
* Grandiflores *	*D. apetala*, *D. firmula*, *D. glaberrima*, *D. grandiflora*, *D. monticola*, *D. ovata*, *D. paposana, D. rotundifolia*
* Holosteoides *	*D. holosteoides, D. pachyphylla*
* Laxiflores *	*D. laxiflora*
* Leptophylla *	*D. effusa, D. leptophylla*
* Lyropetala *	*D. coahuilana*, *D. elata*, *D. lyropetala*, *D. pratheri*, *D. subumbellata, D. suffruticosa*
* Ortegioides *	*D. ortegioides*
* Stipitatae *	*D. stipitata*
* Tenues *	*D. anomala, D. tenuis*
* Villosae *	*D. conzattii*, *D. malachioides*, *D. multiflora, D. villosa*
* Viscosae *	*D. viscosa*

### ﻿Nomenclator, distribution, notes, and new type designations

#### ﻿1. Drymaria

Taxon classificationPlantaeCaryophyllalesCaryophyllaceae

Willd. in Roem. & Schult., Syst. Veg. 5: 406. 1819. Type (designated by Fosberg 1945, pg. 96): Drymaria cordata (L.) Willd.

644B4AF6-149D-534E-8455-18DB5508836D

Pinosia Urb., Arkiv för Botanik 23A(5): 70. 1930. Type: Pinosa ortegioides (Griseb.) Urb. (=Drymaria ortegioides Griseb.)
Mollugophytum M.E.Jones, Contr. W. Bot. 18: 35 (1933). Type, designated here: Mollugophytum holosteoides (Benth.) M.E. Jones (=Drymaria holosteoides Benth.)

##### Notes.

Although authorship of *Drymaria* is often given as “Willd. ex Schult.,” the name and description occur in a single paragraph followed by “Reliq. Willd. MS.” This represents direct association of Willdenow with both the name and the description, and Willdenow should be cited as author (see Turland et al. (2018), art. 46.3 example 15). *Drymaria arenarioides* has been stated to be the type of *Drymaria* (e.g., Duke 1961, Hernández-Ledesma et al. 2015), with Britton and Millspaugh (1920) credited with having made the designation. However, according to Richard Rabeler (pers. comm. 2025), Britton and Millspaugh followed the largely mechanical method of choosing the first species as the type, and their choice is superseded by Fosberg’s designation of *Drymaria cordata* as the type (see Turland et al. (2018) art. 10.7), which was made in the context of discussing morphological characters to evaluate the status of *Mollugophytum* and did not follow the mechanical method.

*Mollugophytum holosteoides* is here designated the type because it is the most widespread species of those treated by Jones (1933) within *Mollugophytum*.

#### ﻿1. Drymaria anilii

Taxon classificationPlantaeCaryophyllalesCaryophyllaceae

Sindhu Arya, Harsh Sing & Iamonico, Plants 13: 3378. 2024.

9F96B363-88CA-5031-9B0A-1BB68BCAB9BD

##### Type.

India. Nagaland: Kohima District, Kisama Village, 25°37'35"N, 94°60'48.9"E, alt. 1800 m, 20 October 2021, *Arya 730* (holotype DMP n.v.; isotypes DMP n.v., RO n.v.).

##### Distribution.

India.

##### Notes.

We suspect that these plants are hybrids between Drymaria cordata subsp. diandra and *D. villosa*; they require more study. This species is assessed as data deficient (DD) (Arya et al. 2024).

#### ﻿2. Drymaria anomala

Taxon classificationPlantaeCaryophyllalesCaryophyllaceae

S.Watson, Proc. Amer. Acad. Arts 25: 143. 1890.

4303B7C6-DE19-5958-99A2-2BA38EDAFA06

##### Type.

Mexico. Coahuila: Carneros Pass, 9 September 1889, *C. G. Pringle 2847* (holotype GH [00037715 image!]).

##### Distribution.

Mexico.

##### Notes.

Duke (1961) mentioned that the holotype of *Drymaria anomala* is deposited in the US. However, this statement is an error, and the holotype is in fact the sheet at GH, where Sereno Watson worked. According to Eric Schuettpelz (pers. comm. 2024), the curator of the US, there are no specimens of *C.G. Pringle 2847* in their herbarium.

#### ﻿3. Drymaria apetala

Taxon classificationPlantaeCaryophyllalesCaryophyllaceae

Bartl. in Presl, Reliq. Haenk. 2(1): 7. 1831.

8C103D7E-4A20-598A-A3D9-B2EE6662D336

=Stellaria virgata Ser. ex DC., Prodr. 1: 396. 1824. Type: Without data (holotype G [G00212357!]).
=Drymaria macrantha A.Gray, U. S. Expl. Exped. 126. 1854. Type: Peru. [Lima]: Cobradillo [illegible, possibly misspelled as Obrajillo, town located in Canta, Lima], 1838, *Wilkes Exploring Expedition s.n.* (lectotype, designated by Duke 1961, pg. 234: US [10417/00103348!]; isolectotype: GH [00037755 image!]).
=Drymaria virgata Briq., Annuaire Conserv. Jard. Bot. Genève 13: 370. 1911. Type: Peru (erroneously reported as Mexico in the protologue). Without data, *H. Ruiz & J. Pavon s.n.* (holotype: G [G00226765 image!]).

##### Type.

Chile. Habitat in Chile, 1822, *T. Haenke s.n.* (lectotype, designated here: GOET [GOET000576 image!]; isolectotypes: HAL [HAL0117876 image!], PR [24309 image!]).

##### Distribution.

Peru, Chile.

##### Notes.

In most cases, for new species described in Reliquiae Haenkeanae, the specimens in PR are considered holotypes. However, in the case of the treatment of Caryophyllaceae, which was prepared by Friedrich Gottlieb Bartling, there are also duplicates housed in GOET, of the Georg August Universität Göttingen. This is the institution where Bartling worked, and these specimens were likely available for study by him at the time of the species’ description. What we are uncertain of is whether he also examined the material at PR. Because of this doubt, we designated the specimen at GOET as the lectotype.

For *Drymaria macrantha*, Duke stated that the specimen in the US was the “HOLOTYPE.” However, both the specimen at US and that at GH were available to Asa Gray at the time of description and labeled by him as a new species. Thus, we treat Duke’s use of the term holotype as an error to be corrected into a lectotype (see Turland et al. (2018) art. 9.10).

The type of *Drymaria virgata* was reportedly collected by Pavon in Mexico, but these are probably duplicates from a collection in Peru, since there are no known specimens collected outside of Peru (Duke 1961), and Ruiz and Pavon did not collect in Mexico.

#### ﻿4a. Drymaria arenarioides Willd. in Roem. & Schult., Syst. Veg. 5: 406. 1819. subsp. arenarioides

Taxon classificationPlantaeCaryophyllalesCaryophyllaceae

F0089F01-CE8A-586D-A33A-A7EF9FAECC86

≡Drymaria frankenioides Kunth, Nov. Gen. Sp. 6 [folio]: 18; [quarto]: 21; tab. 515. 1823. *nom. illeg. superfl.*

##### Type.

Mexico. [Hidalgo]: Pachuca, [May 1803], *A. von Humboldt & A. J. A. Bonpland 4070* (lectotype, designated here: P [P00679593!]; isolectotype: HAL [HAL0117875 image!]).

##### Distribution.

Mexico.

##### Notes.

Although authorship of *Drymaria arenarioides* is often given as “Willd. ex Schult.,” the name and description occur in a single paragraph followed by “Reliq. Willd. MS.” This represents direct association of Willdenow with both the name and the description, and Willdenow should be cited as author (see Turland et al. (2018), art. 46.3 example 15). Because the holotype in B was destroyed (Duke 1961; Hiepko 1987), we here designate the P specimen as the lectotype.

#### ﻿4b. Drymaria arenarioides subsp. peninsularis

Taxon classificationPlantaeCaryophyllalesCaryophyllaceae

(S.F.Blake) J.A.Duke, Ann. Missouri Bot. Gard. 48 (3): 198. 1961.

229BA077-FD0A-505A-8705-78727FF7D422

≡Drymaria peninsularis S.F.Blake, J. Wash. Acad. Sci. 14(13): 285. 1924. Type: Mexico. Baja California Sur: Cape Region, Jan.–Mar. 1901, *C. A. Purpus 423* (holotype: US [470422/00103356 image!]; isotypes: UC [135816/UC 135816 image!], MO [3343597/144084 image!]).
=Drymaria johnstonii Wiggins, Proc. Calif. Acad. Sci. ser. 4, 25: 203. 1944. Type: Mexico. Baja California Sur: in crevices of rock on mesa near crest of island, the isthmus, Espiritu Santo Island, 31 May 1921, *I. M. Johnston 3972* (holotype: CAS [82814/0002334 image!]).

##### Distribution.

Mexico.

#### ﻿5. Drymaria auriculipetala

Taxon classificationPlantaeCaryophyllalesCaryophyllaceae

Mattf., Notizbl. Bot. Gart. Berlin-Dahlem 13(118): 438. 1936.

D645789C-AD04-5FBE-8A52-617290C4C4AC

##### Type.

Peru. Huánuco: Llata, alt. 2500 m, 21 August 1922, *J. F. Macbride & W. Featherstone 2264* (holotype: F [518748/F0042709F image!]; isotypes: GH [00037748 image!], MO [1186085/492968 image!], NY [00342514 image!], US [00103329 image!]).

##### Distribution.

Peru.

##### Notes.

The sheet at F was labeled by Duke as an isotype, but it is in fact the holotype. It was correctly cited as the holotype in Duke (1961). Currently, this species is listed as endangered (EN) (Cano and Sánchez 2006) with occurrences in C Peru.

#### ﻿6. Drymaria axillaris

Taxon classificationPlantaeCaryophyllalesCaryophyllaceae

Brandegee, Univ. Calif. Publ. Bot. 4(11): 178. 1911.

BEE4A094-0309-56F9-8896-8A80448FC36F

##### Type.

Mexico. Coahuila: Sierra del Rey, June 1910, *C. A. Purpus 4526* (holotype: UC [144769/UC 144769 image!]; isotypes: E [E00326061 image!], F [344117/F0053286F image!], GH [00037716 image!], MO [3343598/144085 image!], US 841897/00103330 image!]).

##### Distribution.

United States, Mexico.

#### ﻿7. Drymaria barkleyi

Taxon classificationPlantaeCaryophyllalesCaryophyllaceae

J.A.Duke & Steyerm., Ann. Missouri Bot. Gard. 48(3): 199. 1961.

6E32E8B0-8AB5-5F5F-8D3E-E39C7085C8B8

##### Type.

Mexico. Coahuila: 25 miles southwest of Monterrey, 1 December 1945, *B. H. Warnock & F. A. Barkley 14826* (holotype: F [1176679/F0053280F image!]; isotypes: GH [00037717 image!], TEX [00370752 image!]).

##### Distribution.

Mexico.

#### ﻿8. Drymaria coahuilana

Taxon classificationPlantaeCaryophyllalesCaryophyllaceae

(I.M.Johnst.) B.L.Turner, Phytologia 78(3): 200. 1995.

1115BB0B-D44B-56C8-A663-61E6B7801724

≡Drymaria lyropetala var. coahuilana I.M.Johnst., J. Arnold Arbor. 31: 189. 1950. Type: Mexico. Coahuila: 2 km NW of St. Elena. Vicinity of Santa Elena Mines, eastern foothills of the Sierra de las Cruces, 21 June 1941, *R. M. Stewart 567* (holotype: GH [00037732 image!]; isotypes: ANSM [ANSM007430 image!], BM [BM000522057 image!], DUKE [310217/10000517 image!], F [1757720/F0053298F image!], LL [299864/00370754 image!], MEXU [204377/ MEXU00204377!], UC [1416791/UC 1416791 image!]).

##### Distribution.

Mexico.

#### ﻿9. Drymaria conzattii

Taxon classificationPlantaeCaryophyllalesCaryophyllaceae

J.A.Duke, Ann. Missouri Bot. Gard. 48(3): 224. 1961.

45AFA79A-6FEF-51D1-8907-085734CDCC0D

##### Type.

Mexico. Oaxaca: from Almoloya to Sta. Catarina, alt. 1000 m, 26 December 1906, *C. Conzattii 1688* (holotype: MO [839425/144086 image!]; isotypes: US [572151/00103335 image!], MEXU [MEXU00528401!]).

##### Distribution.

Mexico.

#### ﻿10a. Drymaria cordata (L.) Willd. in Roem. & Schult., Syst. Veg. 5: 406. 1819. subsp. cordata

Taxon classificationPlantaeCaryophyllalesCaryophyllaceae

D8F73D76-1C08-555C-ABA5-5761511AF3CF

≡Holosteum cordatum L., Sp. Pl. 1: 88. 1753. Type: Tab. 11 “Alsine americana nummulariae folio” in Hermann, Paradisus Batavus, 1698 (lectotype, designated by Burger in Cafferty and Jarvis 2004, pg. 1052).
=Holosteum diandrum Sw., Prod. 27. 1788. Type: Jamaica. s.d., *O. Swartz s.n.* (holotype: S [S10-22103 image!]; isotypes: G [G00215840 image!], S [S-R-2936 image!]).
≡Drymaria diandra (Sw.) Macfad., Fl. Jamaica 1: 52. 1837. *nom. illeg*. [non D. diandra Blume (1825)].
≡Drymaria cordata var. diandra (Sw.) Griseb., Fl. Brit. W. I. 56. 1859.
=Drymaria cordata var. puberula Triana & Planch., Ann. Sci. Nat., Bot., sér. 4, 17: 148. 1862. Type: Colombia. [Cundinamarca]: Bogota, alt. 2600 m, s.d., J. J. Triana s.n. (lectotype, designated by Duke 1961, pg. 251: P n.v.; isolectotype NY [3578908 image!]).
=Drymaria procumbens Rose, Contr. U.S. Natl. Herb. 1(9): 304. 1895. Type: Mexico. Colima: near water ditches about Colima, 9 Jan.–6 Feb. 1891, *E. Palmer 1165* (holotype: US [15185/00103359 image!]; isotypes: K [K000471743 image!], NY [00342506 image!], US [1391060/00955563 image!]).
=Drymaria adenophora Urb., Repert. Spec. Nov. Regni Veg. 21(8–20): 213. 1925. Type: Cuba. Pinar del Río: Cortez, 27 March 1921, *L. E. Ekman 12798* (**lectotype**, **designated here**: S [S-R-1684 image!]; isolectotypes: G [G00226766 image!], NY [00073724 image!]).
≡Stellaria adenophora (Urb.) Leon, Contr. Ocas. Mus. Hist. Nat. Colegio “De La Salle” 9: 4. 1950.
= Drymaria sessilifolia Fiori, Anal Bot. Fieni Imp.: 20. 1939. Type: Ethiopia. Adis Abeba, Olettà, alt. 1500 m, 19 October 1937, *E. Bartolozzi 13* (holotype: FT [FT001060 image!]).
=Drymaria cordata var. pacifica M.Mizush., J. Jap. Bot. 32(3): 78. 1957. Type: Ecuador. Galapagos Islands: Academy Bay, Indefatigable Islands, 100 ft [alt. 30.48 m], 1 April 1930, *H. K. Svenson 65* (holotype: GH [00037749 image!]).

##### Distribution.

Subspecies of wide distribution, from the United States to South America and the Caribbean.

##### Notes.

Mizushima (1957) chose the specimen at Herb. Linn. no. 109-1 as the lectotype of *Drymaria cordata*. However, this sheet, as mentioned by Burger in Cafferty and Jarvis (2004), is a post-1753 addition to the Linnaean herbarium and consequently cannot be considered original material and is ineligible for typification of the name.

Although the illustration designated as type is not of high quality in showing some details of the flowers, e.g., the petals appear undivided, there is no doubt that it corresponds to the concept of Drymaria cordata subsp. cordata as currently recognized.

There are two sheets of *Drymaria diandra* at S. The specimen considered here as an isotype is apparently a fragment of the holotype that was later removed from the specimen.

Regarding the type of Drymaria cordata var. puberula, Duke (1961) used the term holotype for the *Triana* specimen at P cited above; however, various syntypes were mentioned in the protologue without a formal designation of type. Thus, Duke’s statement should be treated as an error to be corrected to a lectotype.

Most of the type specimen deposited at B were destroyed in World War II (Hiepko 1987). Because the holotype in B of *Drymaria adenophora* was destroyed, we here designated the specimen at S as the lectotype.

For the same reason as with *Drymaria arenarioides*, authorship of the *Drymaria cordata* combination should be attributed to Willdenow (see Turland et al. (2018), art. 46.3 example 15).

#### ﻿10b. Drymaria cordata subsp. diandra

Taxon classificationPlantaeCaryophyllalesCaryophyllaceae

(Blume) J.A.Duke, Ann. Missouri Bot. Gard. 48(3): 253. 1961.

C5A6EF59-AA0F-5654-B705-E2EE2C4DA6D6

≡Drymaria diandra Blume, Bijdr. Fl. Ned. Ind. (2): 62. 1825. Type: Indonesia. Java: according to the protologue, “in paludosus montanis Javae insulae,” s.d., *C. L. Blume 1549* (lectotype, designated by Mizushima 1957, pg. 81: L [L 0038673 image!]; isolectotypes: L [L 0038674 image!], MO [1765827/216778 image!]).
=Drymaria retusa Wight & Arn., Prodr. Fl. Ind. Orient. 359. 1834. *nom. invalid.* Type: India. Peninsula Ind. Orientalis, s.d., *R. Wight 152* (holotype: G [G00226767 image!]).
=Drymaria extensa Wall., Fl. Brit. India 1(2): 244. 1874. *nom. invalid.* Type: India. Ind. Orient., 1829, *N. Wallich 647* (holotype: G [G00226769 image!]).
=Drymaria gerontogea F.Muell., Descr. Notes Papuan Pl. 1(5): 87. 1877. *nom. invalid.*

##### Distribution.

Subspecies of wide distribution throughout Africa, Asia, and Oceania.

##### Notes.

*Drymaria retusa* and *Drymaria extensa* are invalid names because they were merely cited as provisional synonyms of *D. cordata* and not accepted. *Drymaria gerontogea* has been considered validly published by various authors (e.g., Duke, 1961). However, it was not definitively accepted at the time of publication. Mueller merely states, “Should further research prove Blume’s and Macfadyen’s plants distinct, then I would propose the species name *D. gerontogea* for the former.”

#### ﻿11. Drymaria cubana

Taxon classificationPlantaeCaryophyllalesCaryophyllaceae

Alain, Phytologia 8: 369. 1962.

CA68CE82-A339-5452-919F-86463E72B4B9

≡Pinosia glandulosa Alain, Candollea 17: 107. 1960. Type: Cuba. Oriente: Farallón de Macambo, Vía Azul, km. 70, 28 December 1954, *Alain & López Figueiras 4202* (holotype: LS n.v.).

##### Distribution.

Cuba.

##### Notes.

When Liogier (1962) transferred *Pinosia glandulosa*, the epithet “glandulosa” could not be used for this species due to the existence of *Drymaria glandulosa* C. Presl. He proposed the new name *Drymaria cubana*.

#### ﻿12. Drymaria debilis

Taxon classificationPlantaeCaryophyllalesCaryophyllaceae

Brandegee, Proc. Calif. Acad. Sci., ser. 2, 2: 131. 1889.

6A3B6F9F-B830-53CC-BE54-8D4B1C74131E

=Drymaria polystachya Brandegee, Zoë 2(1): 70. 1891. Type: Mexico. Baja California Sur: San José del Cabo, 24 September 1890, *T. S. Brandegee 35* (holotype: UC [120336/UC 120336 image!]; isotypes: NY [00342505 image!], POM [151790/0002392 image!], US [10418/00103357 image!, 735811/00931426 image!]).
=Drymaria diffusa Rose, Contr. U.S. Natl. Herb. 1(5): 130, pl. 12. 1892. Type: Mexico. Baja California Sur: Carmen Island, 1 November 1890, *E. Palmer 819* (holotype: US [10399/00952505 image!]; isotypes: F [166343/F0053289F image!], GH [00311114 image!], LE [LE 00001741 image!], UC [120326/UC 120326 image!], US [1391051/00952506 image!]).
≡Drymaria polystachya var. diffusa (Rose) Wiggins, Proc. Calif. Acad. Sci., ser. 4, 25: 198. 1944.

##### Type.

Mexico. Baja California Sur: Purisima, 13 February 1889, *T. S. Brandegee s.n.* (holotype: UC [120315/UC 120315 image!]; isotypes: CAS [0002340 image!], PH [01031254/00009747 image!], US [10402/00103336 image!]).

##### Distribution.

Mexico.

##### Notes.

The isotype at CAS of *Drymaria debilis* represents fragments removed from the holotype.

#### ﻿13a. Drymaria divaricata

Taxon classificationPlantaeCaryophyllalesCaryophyllaceae

Kunth, Nov. Gen. Sp. 6 [folio]: 20; [quarto]: 24. 1823. var. divaricata

B030AA1C-FCD4-53C0-AD37-B8C046CFD259

=Drymaria peruviana Muschl., Bot. Jahrb. Syst. 45: 451. 1911. Type: Peru. “la Oroya, in departimento Junin, in formatione plantis pulvinaribus et caespitosis composita, 4300 m s. m.,” February 1903, *Weberbauer 2601* (syntype B, destroyed); Departimento Junin, in montibus prope Tarma in formatione aperta plantis herbaceis (praesertim graminibus) et fruticibus composita, 3600–3700 m s. m.,” 10 February 1903, *Weberbauer 2411* (syntype B, destroyed); “Departimento Junin, La Oroya, in sabulosis, 3900 m s. m.,” February 1903, *Weberbauer 2589* (syntype B, destroyed); Pocarà, statio viae ferreae, in saxosis, 3700 m s. m.,” 24–25 February 1902, *Weberbauer 400* (syntype B, destroyed).

##### Type.

Peru. Lima: crescit ad litora Oceani Pacifici, [1802], *A. von Humboldt & A. J. A. Bonpland s.n.* (holotype: P [P00679596!]).

##### Distribution.

Peru, Bolivia.

##### Notes.

Most of the type specimen deposited at B were destroyed in World War II (Hiepko 1987). The four collections cited in the protologue of *Drymaria peruviana* were apparently destroyed, because they are no longer at B. We have not been able to locate any duplicates.

#### ﻿13b. Drymaria divaricata var. stricta

Taxon classificationPlantaeCaryophyllalesCaryophyllaceae

(Rusby) J.A.Duke, Ann. Missouri Bot. Gard. 48(3):242. 1961.

5322387E-228B-5033-8FB1-ECAE0D3CD66E

≡Drymaria stricta Rusby, Phytologia 1: 55. 1934. Type: Bolivia. [La Paz]: Pongo, 12000 ft [alt. 3657.6 m], 17 Feb.–1 Mar. 1926, *G. H. H. Tate 160* (holotype: NY [00342520 image!]).
=Drymaria agapatensis Baehni & J.F.Macbr., Publ. Field Mus. Nat. Hist., Bot. Ser. 13(2/2): 619. 1937. Type: Peru. [Ayacucho]: Agapata, January 1854, *W. Lechler 1947* (holotype: G [G00226770 image!]; isotypes: L [L 00012078 image!], S [S-R-1685 image!]).

##### Distribution.

Peru, Bolivia.

#### ﻿13c. Drymaria divaricata var. viscidula

Taxon classificationPlantaeCaryophyllalesCaryophyllaceae

(A.Gray) J.A.Duke, Ann. Missouri Bot. Gard. 48(3): 243. 1961.

A1740BA5-F306-579A-8C4C-5860891752A6

≡Drymaria viscidula A.Gray, U. S. Expl. Exped. 124. 1854. Type: Peru. [Lima]: Obrajillo, 1838, *Wilkes Exploring Expedition s.n.* (holotype: US [10427/00103369 image!]).

##### Distribution.

Peru.

#### ﻿13d. Drymaria divaricata var. divergens

Taxon classificationPlantaeCaryophyllalesCaryophyllaceae

J.A.Duke, Ann. Missouri Bot. Gard. 48(3): 243. 1961.

F9468135-0442-519E-A0F6-E4983E2F331D

##### Type.

Peru. Lima: Pativilca, lomas de Pacar, alt. 150–700 m, 1 October 1949, *O. V. Nuñez 2320* (holotype: US [2248528/00603655 image!]; isotype: MO [2174615/491441 image!]).

##### Distribution.

Peru.

#### ﻿13e. Drymaria divaricata var. reflexiflora

Taxon classificationPlantaeCaryophyllalesCaryophyllaceae

J.A.Duke, Ann. Missouri Bot. Gard. 48(3): 244. 1961.

82F4E138-D15D-5441-B458-895880C6A223

##### Type.

Peru. Lima: Chancay, lomas de Lachay, alt. 500–600 m, 5 November 1955, *R. Ferreyra 11522* (holotype: MO [2174617/492967 image!]; isotype: USM [18367/USM000298 image!]).

##### Distribution.

Peru.

#### ﻿14a. Drymaria effusa A.Gray, Smithsonian Contr. Knowl. 5(6): 19. 1853. var. effusa

Taxon classificationPlantaeCaryophyllalesCaryophyllaceae

37E8F6F4-E589-546E-8FE4-EEC8AE1DA9E2

##### Type.

Mexico. Sonora: mountains east of Santa Cruz, 28 September 1851, *C. Wright 869* (holotype: GH [00037671 image!]; isotypes: G [G00226771 image!], GH [00254586 image!], MO [3578826/216548 image!], PH [01031255/00009749 image!]).

##### Distribution.

United States, Mexico.

#### ﻿14b. Drymaria effusa var. confusa

Taxon classificationPlantaeCaryophyllalesCaryophyllaceae

(Rose) J.A.Duke, Ann. Missouri Bot. Gard. 48(3): 205–206. 1961.

4EFCBEDF-14D3-5533-B070-BFF6313068B2

≡Drymaria confusa Rose, Contr. U. S. Natl. Herb. 5 (8): 133. 1897. Type: Mexico. Chihuahua: San José, Southwestern Chihuahua, [28°37'N, 106°04'W], 1885, *E. Palmer 59* (holotype: US [129525/00103333 image!]; isotypes: GH [00037720 image!], K [K000471741 image!], LE [LE 00001742 image!], MO [1807078/144088 image!], VT [UVMVT0224423 image!]).

##### Distribution.

Mexico.

#### ﻿14c. Drymaria effusa var. depressa

Taxon classificationPlantaeCaryophyllalesCaryophyllaceae

(Greene) J.A.Duke, Ann. Missouri Bot. Gard. 48(3): 206–208. 1961.

1720F9E3-4B54-5440-B070-344229A325F7

≡Drymaria depressa Greene, Leafl. Bot. Observ. Crit. 1(11): 153. 1905. Type: United States. New Mexico: around the south end of the Black Range. Sawyer’s Peak. Grant County, [32°47'N, 108°23'W], 9500 ft [alt. 2895.6 m], 30 September 1904, *O. B. Metcalfe 1430* (**lectotype, designated here**: MO [3578825/216549 image!]; isolectotypes: AC [10967/00319675 image!], CAS [82826/0033361 image!], COLO [0363028 image!], E [E00318138 image!], F [187730/F0053281F image!], GH [00037670 image!], NY [00342487 image!], MSC [193377/MSC0091647 image!], RM [90033/RM0002158 image!], POM [151505/0002397 image!], UNM [295/UNM00039 image!]).
=Drymaria minuscula Standl. & Steyerm., Publ. Field Mus. Nat. Hist., Bot. Ser. 23(2): 52. 1944. Type: Guatemala. Huehuetenango: Vicinity of Chemal, summit of Sierra de Los Cuchumatanes, 8 August 1942, *J. A. Steyemark 50243* (holotype: F [1129120/F0053300F image!]; isotype: US [1920024/00103349 image!]).

##### Distribution.

United States, Mexico, Guatemala.

##### Notes.

No herbaria were cited by Greene in the protologue for *Drymaria depressa*. Duke (1961) reported that the holotype was at ND, presumably because that is where Greene worked at the time he described *D. depressa*. According to Barbara Hellenthal (pers. comm. 2024), the curator of ND, there are no specimens of Metcalfe 1430 in their herbarium, and it is likely that the material used by Greene was subsequently returned to Metcalfe. In the absence of the holotype, we here designate the specimen at MO as the lectotype because it is the most complete.

#### ﻿15. Drymaria elata

Taxon classificationPlantaeCaryophyllalesCaryophyllaceae

I.M.Johnst., J. Arnold Arbor. 21: 68. 1940.

76A2B6C5-3B75-506E-9D3D-2F159D750DEC

##### Type.

Mexico. Coahuila: road from Mohovano northeast to Estancia Station: 6 miles south of Laguna del Rey, 21 September 1938, *I. M. Johnston 7823* (holotype: GH [00037722 image!]).

##### Distribution.

Mexico.

#### ﻿16a. Drymaria engleriana (Muschl.) Baehni & J.F.Macbr., Publ. Field Mus. Nat. Hist., Bot. Ser. 13(2/2): 621. 1937. var. engleriana

Taxon classificationPlantaeCaryophyllalesCaryophyllaceae

66F522FC-262B-5770-B0CC-C8A34E2D47D0

≡Polycarpon englerianum Muschl., Bot. Jahrb. Syst. 45(4): 452. 1911. Type: Peru. Ancash: Caraz, alt. 4300 m, 1906, *A. Weberbauer 3101* (**lectotype, designated here**: MOL [00219/00000486 image!]; isolectotype: G [G27707/00226772 image!]).

##### Distribution.

Peru.

##### Notes.

Most of the type specimen deposited at B were destroyed in World War II (Hiepko 1987). Because the holotype in B was probably destroyed, we here designate the MOL specimen as the lectotype. Currently, this variety is listed as Endangered (EN) (Cano and Sánchez 2006).

#### ﻿16b. Drymaria engleriana var. devia

Taxon classificationPlantaeCaryophyllalesCaryophyllaceae

(Baehni & J.F.Macbr.) J.A.Duke, Ann. Missouri Bot. Gard. 48(3): 219. 1961.

DF4A4FF6-BC70-561B-96F8-C418407EE32C

≡Drymaria devia Baehni & J.F.Macbr., Publ. Field Mus. Nat. Hist., Bot. Ser. 13(2/2): 620. 1937. Type: Peru. Lima: near Antaicocha, Cerro Colorado, east of Canta, alt. 3800–4000 m, 20 June 1925, *F. W. Pennell 14655* (holotype: F [558629/F0042710F!]; isotypes: GH [00037750 image!], PH [774610/00009748 image!], S [S-R-1686 image!], US [1340894/00103339 image!]).

##### Distribution.

Peru, Chile.

##### Notes.

The sheet at F was labeled by Duke as an isotype, but it is in fact the holotype as cited in Duke (1961).

#### ﻿17. Drymaria excisa

Taxon classificationPlantaeCaryophyllalesCaryophyllaceae

Standl., Publ. Field Mus. Nat. Hist., Bot. Ser. 8(1): 11. 1930.

4C7BCC8F-E307-551C-8A32-52A4368A8139

=Drymaria grandis Bullock, Bull. Misc. Inform. Kew 1936(6): 389. 1936. Type: Mexico. México: Temascaltepec, alt. 2440 m, 21 January 1934, *G. B. Hinton 5427* (holotype: K [K000471758 image!]; isotypes: BM [BM000939014 image!], G [G00226773 image!], GH [00037728 image!], NY [00342498 image!], NY [00342499 image!], RSA [350580/RSA0002395 image!], S [S-G-7134/7134 image!]).

##### Type.

Mexico. Jalisco: Sierra Madre Occidental, Real Alto, trail to El Tajo de Santiago, alt. 2500 m, 23 February 1927, *Y. E. J. Mexia 1748* (holotype: F [579891/F0053290F image!]; isotypes: CAS [157122/0002333 image!], CAS [195467/0027349 image!], G [G00226774 image!], GH [0003724 image!], MICH [1115007 image!], MO [954774/144089 image!], NY [00342496 image!], UC [350610/UC 350610 image!]).

##### Distribution.

Mexico.

#### ﻿18. Drymaria fasciculata

Taxon classificationPlantaeCaryophyllalesCaryophyllaceae

A.Gray, U. S. Expl. Exped., Phan.: 125. 1854.

6BF2E039-C570-56A0-BFAA-3856E8A30131

##### Type.

Peru. [Lima]: Andes near Obrajillo, 1838, *Wilkes Exploring Expedition s.n.* (lectotype, designated by Duke 1961, pg. 217: US [10405/00103342 image!]; isolectotype GH [00037751 image!]).

##### Distribution.

Peru.

##### Notes.

Duke stated that the specimen in the US was the “HOLOTYPE.” However, both the specimen at US and that at GH were available to Asa Gray at the time of description and labeled by him as a new species. Thus, we treat Duke’s use of the term holotype as an error to be corrected to lectotype.

#### ﻿19. Drymaria firmula

Taxon classificationPlantaeCaryophyllalesCaryophyllaceae

Steyerm., Publ. Field Mus. Nat. Hist., Bot. Ser. 28(1): 227. 1951.

9877251A-44C8-5A4B-B27B-99847DF7565A

##### Type.

Venezuela. Merida: Páramo de Los Colorados, between El Molino and San Isidro Alto, alt. 2745–2955 m, 14 May 1944, *J. A. Steyermark 56536* (holotype F: [1340308/0053307F image!]; isotypes: MO [2174628/216513 image!], NY [00342515 image!]).

##### Distribution.

Venezuela.

#### ﻿20. Drymaria frutescens

Taxon classificationPlantaeCaryophyllalesCaryophyllaceae

Mattf., Notizbl. Bot. Gart. Berlin-Dahlem 13(118): 439. 1936.

ACCE5D9D-434E-5577-80E5-180E2E0772E4

##### Type.

Peru. La Libertad: Santiago de Chuco, above Hacienda Angasmarca, alt. 3650 m, May 1916, *A. Weberbauer 7203* (**lectotype, designated here**: F [628040/F0042712F image!]; isolectotypes: F [549079/F0042713F image!], GH [00037752 image!], MO [1768271/216512 image!], US [1495416/00103344 image!]).

##### Distribution.

Peru.

##### Notes.

Duke mentions that the holotype was in B. However, the protologue (Mattfeld, 1936) clearly states that the type is in F. Due to the existence of two sheets at F, here we designated the specimen [628040/0042712F] as the lectotype. This species is listed as Critically Endangered (CR) (Cano and Sánchez 2006).

#### ﻿21. Drymaria glaberrima

Taxon classificationPlantaeCaryophyllalesCaryophyllaceae

Bartl. in Presl, Reliq. Haenk. 2(1): 7. 1831.

0C9AE0ED-2FA2-50A1-B5B5-8ADA7C774828

##### Type.

Peru. [Lima]: Habitat in Peruviae montanis huanoccensibus, 1822, *T. Haenke s.n.* (**lectotype, designated here**: GOET [GOET000577!]; isolectotype: PR [24305 image!]).

##### Distribution.

Peru.

##### Notes.

For the same reason as mentioned for *Drymaria apetala*, we designate the specimen in GOET as the lectotype. This species is listed as Endangered (EN) (Cano and Sánchez 2006).

#### ﻿22a. Drymaria glandulosa Phil., Fl. Atacam. 10. 1860. var. glandulosa

Taxon classificationPlantaeCaryophyllalesCaryophyllaceae

D1AD1642-13EB-541F-82BE-2B6CA7B8F5EF

=Drymaria ramosissima Schltdl., Linnaea 12(2): 206. 1838. Type: Mexico. Prope urben Mexico, s.d., *Hegewisch s.n.* (holotype: HAL [HAL0098412 image!]; isotype: CAS [82822/0002331 image!]).
=Drymaria leptoclados Hemsl., Diagn. Pl. Nov. Mexic. 1: 2. 1878. Type: Guatemala. [Guatemala]: camino del Sapote, December 1865, *K. G. Bernoulli 240* (holotype: K [3862/K000471752 image!]; isotypes: G [G00226778 image!], NY [00342500 image!]).
=Drymaria fendleri S.Watson, Proc. Amer. Acad. Arts 17: 328. 1882. Type: United States. New Mexico, 1 January 1847, *A. Fendler 60* (lectotype, designated by Duke 1961, pg. 247: GH [00037672 image!]; isolectotypes: GH [00037673 image!, 00037674 image!], K [K000723291 image!]).
≡Drymaria glandulosa var. fendleri (S.Watson) Fosberg, Contr. U. S. Natl. Herb. 29(2): 96. 1957.
=Drymaria leptoclados var. peruviana Ball, J. Proc. Linn. Soc., Bot. 22: 32. 1885. Type: Peru. [Lima]: Chicla, 21 April 1882, *J. Ball s.n.* (holotype: K [K000471733 image!]).
=Drymaria blasiana M.E.Jones, Contr. W. Bot. 15: 125. 1929. Type: Mexico. Sinaloa: San Blas, 30 January 1927, *M. E. Jones 22845* (holotype: POM [162740/0002399 image!]).
=Drymaria fendleri var. perennis M.E.Jones, Contr. W. Bot. 18: 65. 1933. Type: Mexico: Baja California Sur: Laguna Mountains, La Laguna, 22 September 1930, *M. E. Jones 27050* (holotype: POM [192443/0002396 image!]; isotypes: F [721383/F0053291F image!], MIN [626682/1000875 image!], NY [00342497 image!], UC [522237/UC 522237 image!]).
≡Drymaria glandulosa var. perennis (M.E.Jones) Fosberg, Contr. U. S. Natl. Herb. 29: 96. 1945.

##### Type.

Mexico: habitat in Mexico, s.d., *T. Haenke s.n.* (holotype: PR [24310 image!]).

##### Distribution.

United States, Mexico, Guatemala, Honduras, Peru, Bolivia, Argentina.

##### Notes.

For *Drymaria fendleri*, Duke stated that the specimen in GH was the “HOLOTYPE.” Thus, we treat Duke’s use of the term holotype as an error to be corrected to lectotype.

#### ﻿22b. Drymaria glandulosa var. galeottiana

Taxon classificationPlantaeCaryophyllalesCaryophyllaceae

(Briq.) J.A.Duke, Ann. Missouri Bot. Gard. 48(3): 249. 1961.

2568A2D6-BD81-5529-B726-C9D36253D64B

≡Drymaria galeottiana Briq., Annuaire Conserv. Jard. Bot. Genève 13: 373. 1911. Type: Mexico. Oaxaca: near Oaxaca, 1840, *H. G. Galeotti 4408* (holotype: G [G00226777 image!]; isotypes: K [K3862/000471749 image!], LE [LE 00001740 image!]).

##### Distribution.

Mexico.

#### ﻿23a. Drymaria gracilis Phil., Fl. Atacam. 10. 1860. subsp. gracilis

Taxon classificationPlantaeCaryophyllalesCaryophyllaceae

31D5F152-9612-59AD-943F-FE115BA82A0D

≡Drymaria cordata var. gracilis (Schltdl. & Cham.) Rohrb., Fl. Bras. 14(2): 260. (1872).

##### Type.

Mexico. Veracruz: Jalapam [Xalapa], August 1827, *C. J. W. Schiede & F. Deppe 503* (holotype: HAL [HAL098420/0098420 image!]; isotypes: GOET [GOET000578!], LE [LE 00001739 image!]).

##### Distribution.

Mexico, Guatemala, Honduras, El Salvador.

##### Notes.

Duke mentioned that the holotype of *Drymaria gracilis* was at B, but it is in fact the specimen at HAL, where Schlechtendal’s herbarium is now housed.

#### ﻿23b. Drymaria gracilis subsp. carinata

Taxon classificationPlantaeCaryophyllalesCaryophyllaceae

(Brandegee) J.A.Duke, Ann. Missouri Bot. Gard. 48(3): 247. 1961.

BCB0CF49-B457-51CC-A458-62304E78C31E

≡Drymaria carinata Brandegee, Zoë 2(1): 70. 1891. Type: Mexico. Baja California Sur: Sierra de la Laguna, 21 January 1890, *T. S. Brandegee 34* (holotype: UC [120331/UC 120331 image!]; isotypes: NY [00342491 image!], POM [101189/0002398 image!]).
=Drymaria carinata var. perennis Wiggins, Proc. Calif. Acad. Sci., ser. 4, 25: 197. (1944). Type: Mexico. Baja California Sur: Sierra Laguna, La Laguna, 6000 ft [alt. 1828.8 m], 25 March 1939, *H. S. Gentry 4415* (holotype: DS [263289/0002338 image!]; isotypes: ARIZ [268235/ARIZ-BOT-0005308 image!, 319000/ ARIZ-BOT-0005625 image!], MO [1159044/144090 image!], UC [UC 708997 image!]).

##### Distribution.

Mexico.

#### ﻿24. Drymaria grandiflora

Taxon classificationPlantaeCaryophyllalesCaryophyllaceae

Bartl. in Presl, Reliq. Haenk. 2(1): 7. 1831.

576653AA-661F-5F63-9290-3B979126F520

##### Type.

Peru. [Huánuco]: Habitat in Chile and in Peruviae montanis huanoccensibus, 1822, *T. Haenke s.n.* (**lectotype, designated here**: GOET [GOET000579 image!]; isolectotypes: GOET [GOET000580 image!], HAL [HAL0117872 image!], PR [24308 image!]).

##### Distribution.

Peru.

##### Notes.

For the same reason as mentioned for *Drymaria apetala*, we designate the specimen at GOET as the lectotype. The protologue mentions that this species grows in Chile. In fact, this information is incorrect (Duke 1961). This species is listed as Near Threatened (NT) (Cano and Sánchez 2006).

#### ﻿25a. Drymaria holosteoides Benth., Bot. Voy. Sulphur: 16. 1844. var. holosteoides

Taxon classificationPlantaeCaryophyllalesCaryophyllaceae

D7877E93-FE75-5051-8CF2-8754ED28F9CF

≡Mollugophytum holosteoides (Benth.) M.E.Jones, Contr. W. Bot. 18: 35. 1933.
=Drymaria veatchii Curran, Proc. Calif. Acad. Sci. 2(1): 227. 1888. Type: Mexico. Baja California Sur: Magdalena Island, 1888, *W. E. Bryant s.n.* (holotype: UC [124950/UC 124950 image!]).

##### Type.

Mexico. Baja California Sur: Bay of Magdalena, 1841, *R. B. Hinds s.n.* (holotype K [K000471757 image!]; isotype LE [LE 00001737 image!]).

##### Distribution.

Mexico.

#### ﻿25b. Drymaria holosteoides var. crassifolia

Taxon classificationPlantaeCaryophyllalesCaryophyllaceae

(Benth.) J.A.Duke, Ann. Missouri Bot. Gard. 48(3): 187. 1961.

DC903FA9-59C1-5B33-B046-8ADB00B89129

≡Drymaria crassifolia Benth., Bot. Voy. Sulphur: 16. 1844. Type: Mexico. Baja California Sur: Cape San Lucas, 1841, *R. B. Hinds s.n.* (holotype: K [3862/K000471763 image!]; isotype: LE [LE 00012079 image!]).
≡Mollugophytum crassifolium (Benth.) M.E.Jones, Contr. W. Bot. 18: 35. 1933.

##### Distribution.

Mexico.

#### ﻿26. Drymaria hypericifolia

Taxon classificationPlantaeCaryophyllalesCaryophyllaceae

Briq., Annuaire Conserv. Jard. Bot. Genève 13–14: 369. 1911.

1962C97E-2EC7-5117-BD56-43E71F41AD17

##### Type.

Mexico. Oaxaca: from Lecambre de Yolotepec to Juquila, southwest of Oaxaca, March 1845, *C. Jürgensen 38* (lectotype, designated by Duke 1961, pg. 201: G [G00226746 image!]; isolectotypes: F [869035/F0053294F image!], K [K000471756 image!]).

##### Distribution.

Mexico, Guatemala, Honduras.

##### Notes.

Two collections were cited in the protologue, Jurgensen no. 38 and Galeotti no. 4425, and neither was designated as the type. Duke (1961) stated that the holotype was Jungersen´s collection.

#### ﻿27. Drymaria jenniferae

Taxon classificationPlantaeCaryophyllalesCaryophyllaceae

Villarreal & A.E.Estrada, Brittonia 60: 329. 2008.

63EE7416-0D94-5D6B-8A3E-A8A0DDAF0042

##### Type.

Mexico. Coahuila: mpio. Viesca, E of 5 de Mayo, alt. 1606 m, 30 September 2006, *G. B. Hinton 28498* (holotype: MEXU [1393821/MEXU01393821!]; isotypes: ANSM [ANSM087856/087856 image!], CIIDIR [CIIDIR022559 image!], GBH [GBH028498 image!]).

##### Distribution.

Mexico.

#### ﻿28. Drymaria ladewii

Taxon classificationPlantaeCaryophyllalesCaryophyllaceae

Rusby, Phytologia 1(2): 54. 1934.

06F53144-9A24-5DC9-A9A2-E241CDC45ACB

##### Type.

Bolivia. Nequejahuira: Cordillera Real, 8000 ft [alt. 2438.4 m], 15–24 May 1926, *G. H. H. Tate 652* (lectotype, designated here: NY [00342516 image!]; isolectotype: NY [00342517 image!]).

##### Distribution.

Mexico, Guatemala, El Salvador, Costa Rica, Panama, Peru, Bolivia.

##### Notes.

There are two separate sheets of *Tate 652* at NY, and neither was indicated as the holotype by Rusby. We select the better of the two as the lectotype.

#### ﻿29. Drymaria laxiflora

Taxon classificationPlantaeCaryophyllalesCaryophyllaceae

Benth., Pl. Hartw.: 73. 1841.

DA032823-9CD6-5AC1-922C-055FECAD2A06

=Drymaria chihuahuensis Briq., Annuaire Conserv. Jard. Bot. Genève 13–14: 370. 1911. Type: Mexico. Chihuahua: near Chihuahua, 1 September 1885, *C. G. Pringle 331* (holotype: G [G00226751 image!]; isotypes: ARIZ [319001/ARIZ-BOT-0005626 image!], CAS [94965/0002339 image!], E [E00326059 image!, 00394858 image!], F [104212/F0053287F image!, 870682/0053288F image!], G [G00226752 image!, 00226762 image!], GH [00037719 image!], JE [JE00014348 image!], KFTA [KFTA0000328 image!], LE [LE 00001743 image!, LE 00001744 image!], LECB [LECB0000546 image!], LL [00370753 image!], MEL [MEL2499824 image!, MEL2499825 image!], MEXU [MEXU00148765!], MICH [1115008 image!], NY [00342492 image!, 00342493 image!, 00342494 image!], UC [UC 41993 image!], US [10411/00931458 image!], VT [UVMVT024422 image!]).

##### Type.

Guatemala. Quetzaltenango: In rupibus Sunil, [14°47'N, 91°29'W], 1839, *K. T. Hartweg 523* (holotype: K [K000471754 image!]; isotypes: E [E00326060 image!], F [871784/F0053295F image!, 869007/0053296F image!], FI [021732/005970 image!], G [G00226750 image!, 00226763 image!], K [K000471753 image!], LD [1216996 image!], LE [LE 00012081 image!], MO [6033460/2090484 image!]).

##### Distribution.

United States, Mexico, Guatemala, Bolivia.

##### Notes.

Duke reported that the holotype of *Drymaria laxiflora* is at BM. However, we interpret the holotype to be the specimen at K ex Herbarium Benthamianum.

For *Drymaria chihuahuensis*, there is a sheet at VT marked as holotype. However, as mentioned by Duke (1961), the holotype is at G.

#### ﻿30a. Drymaria leptophylla (Cham. & Schltdl.) Fenzl ex Rohrb., Linnaea 37(2): 195. 1871–1873 [1872]. var. leptophylla

Taxon classificationPlantaeCaryophyllalesCaryophyllaceae

57CD0858-2A1D-5416-9FDB-BA781D16712E

≡Arenaria leptophylla Cham. & Schltdl., Linnaea 5(2): 233. 1830. Type: Mexico. Hidalgo: al E de Pachuca, al lado de la Carretera Federal 105 hacia Mineral del Monte, Mpio. de Mineral de la Reforma, 20°07'41"N, 98°41'51"W, alt. 2689 m, 28 September 2024, *D. Valentín-Martínez & V. W. Steinmann 1204* (**neotype, designated here**: IEB [274928!]).
≡Spergularia leptophylla (Schltdl. & Cham.) G.Don, Gen. Hist. 1: 425. 1831.
≡Spergula leptophylla (Schltdl. & Cham.) D.Dietr., Syn. Plant. 2: 1598. 1840.
=Drymaria tenella A.Gray, Mem. Amer. Acad. Arts, n.s. 4(1): 12. 1849. Type: United States. New Mexico: 8 miles W of Las Vegas, New Mexico, shady places in woodland in the mountain region, 1847, *A. Fendler 56* (holotype: GH [00037680 image!]; isotype: MO [3578827/2196012 image!]).
=Drymaria nodosa var. gracillima Hemsl., Diagn. Pl. Nov. Mexic. 2: 22. 1879. Type: Mexico. San Luis Potosi: Chiefly in the region of San Luis Potosi, 6000–8000 ft [alt. 1828.8–2438.4 m], 1878, C. C. *Parry & E. Palmer 60* (holotype: K [K000471750!]; isotypes: GH [00037735 image!], MO [3343588/144092 image!], NY [00342502 image!], WIS [v 0254955 WIS image!]).
≡Drymaria gracillima (Hemsl.) Rose, Contr. U.S. Natl. Herb. 5: 132. 1897.

##### Distribution.

United States, Mexico.

##### Notes.

Most of the type specimen deposited at B were destroyed in World War II (Hiepko 1987). Because the holotype of *Arenaria leptophylla* deposited in B was destroyed and no duplicates of the original material were found in other herbaria such as HAL, where Schlechtendal’s herbarium is housed, we designated the specimen at IEB as a neotype. The species was described based on *C. J. W. Schiede 511* from “Montis Orizabae,” Veracruz. In the absence of a good quality specimen from near the type locality, we have chosen the above specimen because it corresponds precisely to the application of the name *Drymaria leptophylla* and is from the neighboring state of Veracruz.

#### ﻿30b. Drymaria leptophylla var. cognata

Taxon classificationPlantaeCaryophyllalesCaryophyllaceae

(S.F.Blake) J.A.Duke, Ann. Missouri Bot. Gard. 48(3): 208. 1961.

DFDB7C75-3016-5BCF-A656-845D2160DA0E

≡Drymaria cognata S.F.Blake, J. Washington Acad. Sci. 14: 285. 1924. Type: Mexico. Durango: vicinity of Durango, April 1896, *E. Palmer 912* (holotype: US [304403/00103332 image!]).

##### Distribution.

Mexico.

#### ﻿30c. Drymaria leptophylla var. nodosa

Taxon classificationPlantaeCaryophyllalesCaryophyllaceae

(Engelm.) J.A.Duke, Ann. Missouri Bot. Gard. 48(3): 209. 1961.

C9C83578-E841-5F83-98A1-A9F653C1E79A

≡Drymaria nodosa Engelm., Mem. Amer. Acad. Arts, ser. 2, 4(1): 12. 1849. Type: Mexico. Chihuahua: Cosiquiriadi, s.d., *F. A. Wislizenus s.n.* (holotype: GH [00037734 image!]).
≡Drymaria tenella var. nodosa (Engelm.) Wiggins, Proc. Calif. Acad. Sci., ser. 4, 25: 205. 1944.
=Drymaria gentryi Fosberg, Proc. Biol. Soc. Washington 62: 147. 1949. Type: Mexico. Chihuahua: Rio Mayo, Los Cascarones, 11 September 1936, *H. S. Gentry 2669* (holotype: US [1686921/00103345 image!]; isotypes: ARIZ [319002/ARIZ-BOT-0005627 image!, 319003/0005628 image!], F [862589/F0053293F image!], GH [00037726 image!], MO [1162012/144091 image!], S [S-G-7136 image!], UC [UC 582148 image!]).

##### Distribution.

United States, Mexico.

#### ﻿31. Drymaria longepedunculata

Taxon classificationPlantaeCaryophyllalesCaryophyllaceae

S.Watson, Proc. Amer. Acad. Arts 25: 142. 1890.

81C41D63-3827-51DE-8A41-D4EFBAAD6932

##### Type.

Mexico. Jalisco: cliffs of the barranca, near Guadalajara, 28 November 1888, *C. G. Pringle 2121* (holotype: GH [00037730 image!]; isotypes: UC [UC 120340 image!], VT [UVMVT024424 image!]).

##### Distribution.

Mexico.

#### ﻿32. Drymaria lyropetala

Taxon classificationPlantaeCaryophyllalesCaryophyllaceae

I.M.Johnst., J. Arnold Arbor. 21: 68. 1940.

22C2C12F-24E7-515E-B12F-796816C09346

##### Type.

Mexico. San Luis Potosi: 2 miles south of Cedral, on the road from Matehuala north to San Miguel on the state-boundary, 11 September 1938, *I. M. Johnston 7594* (holotype: GH [00037731 image!]; isotypes: F [1016593/F0053297F image!], S [S-G-7133 image!], UC [UC 741256 image!], US [1823067/00103347 image!]).

##### Distribution.

Mexico.

#### ﻿33. Drymaria malachioides

Taxon classificationPlantaeCaryophyllalesCaryophyllaceae

Briq., Annuaire Conserv. Jard. Bot. Genève 13: 372. 1911.

A8D9E727-2B9F-5130-B6AF-70522DD2AFD7

##### Type.

Mexico. [Michoacán]: Cordillere d’ Ario, Jun.–Oct. 1840, *H. G. Galeotti 4419* (holotype: G [G00226779 image!]; isotypes: MO [1669694/2090290 image!], P [P04936753 image!]).

##### Distribution.

Mexico.

#### ﻿34. Drymaria molluginea

Taxon classificationPlantaeCaryophyllalesCaryophyllaceae

(Schrad.) Didr., Linnaea 29: 738. 1858.

883F4FB3-6614-527E-8356-BD57462C285B

≡Alsine molluginea Lag., Gen. Sp. Pl. 13. 1816. *nom. illeg.* [non A. molluginea Hornem., (1813)]. Type: Without locality information, s.d., *M. Lagasca “1806*” (lectotype, designated by Duke 1961, pg. 194: G-DC [00212545 image!]).
≡Arenaria molluginea Schrad., Index Seminum [Gottingen] 1819: 2. 1819.
≡Arenaria molluginea Ser., Prodr. 1: 400. 1824. *nom. illeg. superfl.* [non A. molluginea Schrad., 1819].
≡Stellaria molluginea (Schrad.) Sweet, Hort. Brit. ed. 2: 56. 1830.
≡Spergularia molluginea (Schrad.) G.Don., Gen. Hist. 1: 425. 1831.
≡Lepigonum mollugineum (Schrad.) Bartl., Index Seminum [Gottingen] 1838: 4. 1838.
=Alsine molluginea Hornem., Hort. Bot. Hafn. 1: 295. 1815[1813]. Type: Cultivated at the Copenhagen Botanical Garden, s.d., *J.W. Horneman s.n*. (holotype: [C image!]).
=Alsine molluginea Mart., Plant. Hort. Erlang. 82. 1814. *nom. illeg.* [non A. molluginea Hornem., (1813)]. Type: Cultivated at the Erlangen Botanical Garden, s.d., *C.F.P. Martius s.n*. (**lectotype, designated here**: BM [BM001024715 image!] [ex. Herb. Roemer]).
=Drymaria sperguloides A.Gray, Mem. Amer. Acad. Arts 4(1): 10. 1849. Type: United Sates. New Mexico, 1847, *A. Fendler 55* (holotype: GH [00037679 image!]; isotypes: MO [3578836/216516 image!]).
≡Mollugophytum sperguloides (A.Gray) M.E.Jones, Contr. W. Bot. 18:35. 1933.

##### Distribution.

United States, Mexico.

##### Notes.

As mentioned above, the name *Alsine molluginea* was proposed independently three times for the same taxon but with different types. Seringe is often credited as first proposing a name in *Arenaria* in 1824. However, five years earlier, Schrader provided a legitimate name in *Arenaria*. Although he did not include a description or diagnosis, his name should be accepted because his citation of “Alsine*Lag.*” constitutes an indirect reference to Lagasca’s description published in 1816. (see Turland et al. (2018), art. 41.3).

Although the specimen of *Alsine molluginea* at G-DC does not provide information, the protologue states that it is based on plants grown in the “H.R.M.” [Real Jardín Botánico de Madrid] from seed gathered in Nueva Hispania by D.D. Sessé [sic] & J.M. Mociño. *Drymaria molluginea* was first described as *Alsine molluginea* by the Danish botanist Jens Wilken Hornemann in an account of the plants found in the Copenhagen Botanical Garden. His morphological description was brief but stated that the species was an annual with divided petals and thick, linear, secund leaves. He also indicated that the habitat of the species is unknown, but that it was introduced in the garden in 1812 from material provided by the Vienna Botanical Garden. The specimen cited above was labeled by Horneman himself as *Alsine molluginea* (handwriting confirmed by Jens Soelberg, C), and is the only original material known. Martius independently described *Alsine molluginea* in his thesis on the plants from the Erlangen Botanical Garden. He stated that they came from the Paris Botanical Garden “h. Par.” The specimen at BM was labeled by Martius (handwriting confirmed by Han Joaquin Esser, M) as “Alsine molluginea mihi enum. horti. Erlangensis.” There are no specimens at M, where most of Martius’ collections can be found, and for that reason, we designate the collection at BM as the lectotype. The reason that we are attributing authorship of the basionym of *Drymaria molluginea* to Schrader and not to Hornemann is that Lagasca’s name was clearly mentioned when the combination to *Drymaria* was made by Didrichsen (1858), and no indication of Hornemann’s earlier proposal of *Alsine molluginea* was provided.

#### ﻿35. Drymaria monticola

Taxon classificationPlantaeCaryophyllalesCaryophyllaceae

Howell, Proc. Calif. Acad. Sci., Ser. 4, 21: 329. 1935.

81A127C9-A918-53D9-A2DD-6712A3F4C69F

##### Type.

Ecuador. Galapagos Islands: Indefatigable Island, 9 May 1932, *J. T. Howell 9243* (holotype: CAS [0002336 image!]; isotypes: B [B 10 0365633!], S [S-R-1687 image!]).

##### Distribution.

Ecuador.

##### Notes.

This species is listed as Critically Endangered (CR) (León-Yáñez et al. 2011).

#### ﻿36. Drymaria multiflora

Taxon classificationPlantaeCaryophyllalesCaryophyllaceae

Brandegee, Zoë 5(11): 232. 1906.

8B059505-C5EE-5E64-845B-4D843F8BA9F4

##### Type.

Mexico. [México]: Salto de Agua, October 1905, *C. A. Purpus 1653* (lectotype, designated by Duke 1961, pg. 224: UC [UC 125379 image!]; isolectotypes: F [193175/F0053301F image!], GH [00037733 image!], MO [3578885/144093 image!], US [570477/00103350 image!]).

##### Distribution.

Costa Rica, Guatemala, Mexico, Panama.

#### ﻿37. Drymaria ortegioides

Taxon classificationPlantaeCaryophyllalesCaryophyllaceae

Griseb., Cat. Pl. Cub.: 21. 1866.

D827170E-29ED-5C38-94E1-A679BB7864B0

≡Pinosia ortegioides (Griseb.) Urb., Ark. Bot. 23A(5): 71. 1930.

##### Type.

Cuba. Cuba occ., 1863, *C. Wright 2019* (holotype: GOET [GOET000581 !]; isotypes: BM [BM000939016 image!], G [G00226747 image!], GH [00037747 image!], K [K000471735 image!], LE [LE 00001735 image!], NY [00073725 image!, 00073726 image!], S [S-R-1688 image!, S-R-1689 image!], US [10421/00103352 image!], YU [YU.001065 image!]).

##### Distribution.

Cuba.

#### ﻿38. Drymaria ovata

Taxon classificationPlantaeCaryophyllalesCaryophyllaceae

Willd. in Roem. & Schult., Syst. Veg. 5: 406. 1819.

A6FDC44C-469A-5C8D-92A5-B31143B310E1

##### Type.

Ecuador. Quito, [1802], *A. von Humboldt & A. J. A. Bonpland s.n.* (holotype: P [P00274219!]; isotype: P [P00335772!]).

##### Distribution.

Ecuador, Venezuela, Peru, Bolivia, Argentina.

##### Notes.

The specimens deposited in P with the label “Herbier Humboldt & Bonpland” come from Bonpland’s private collection. According to Stauffer et al. (2012), these are to be considered holotypes. In the case of *Drymaria ovata*, there are two specimens at P representing original material. The one with the barcode “P00274219” bears the label “Herbier Humboldt & Bonpland” and represents the holotype.

For the same reason as with *Drymaria arenarioides*, authorship of *Drymaria ovata* should be attributed to Willdenow (see Turland et al. (2018), art. 46.3 example 15).

#### ﻿39. Drymaria pachyphylla

Taxon classificationPlantaeCaryophyllalesCaryophyllaceae

Wooton & Standl., Contr. U.S. Natl. Herb. 16(3): 121. 1913.

A1CB1ADF-704C-5246-955B-921761F89F5A

##### Type.

United States. New Mexico: south of White Sands, 4100 ft [alt. 1249.6 m], 28 August 1897, *E. O. Wooton 405* (holotype: US [330629/00103353 image!]; isotypes: CAS [93988/0033360 image!], E [E00318137 image!], G [G00226748 image!, 00226749 image!], LE [LE 00001734 image!], MO [1746734/216515 image!], NDG [49829/16745 image!], NY [00342490 image!], RM [RM0002159 image!], US [735952/00931427 image!]).

##### Distribution.

United States, Mexico.

#### ﻿40a. Drymaria paposana Phil., Fl. Atacam. 10. 1860. var. paposana

Taxon classificationPlantaeCaryophyllalesCaryophyllaceae

A029B3F8-A2C7-5184-BBAA-5146617CFCD1

##### Type.

Chile. [Antofagasta]: Prope Paposo in valle Tartal, s.d., *R. A. Philippi s.n.* (holotype: SGO [048885/SGO000001961 image!]).

##### Distribution.

Bolivia, Chile.

##### Notes.

At MO, there is a photo of an isotype ([048885/166961!]) probably from the Austrian herbarium W.

#### ﻿40b. Drymaria paposana var. weberbaueri

Taxon classificationPlantaeCaryophyllalesCaryophyllaceae

(Muschl.) J.A.Duke, Ann. Missouri Bot. Gard. 48(3): 238. 1961.

4E69DC72-519A-5485-ADC0-939BD34A68DA

≡Drymaria weberbaueri Muschl., Bot. Jahrb. Syst. 45(4): 451. 1911. Type: Peru. [Lima]: Barranca prope Lima in formatione “Loma” dicta, alt. 50–200 m, s.d., *A. Weberbaueri 1662* (lectotype, designated by Duke 1961, pg. 238: G [G00226776 image!]).
=Drymaria fenzliana Briq., Annuaire Conserv. Jard. Bot. Genève 13: 373. 1911. Type: Mexico [Peru]. Without data, *J. A. Pavon s.n.* (holotype: G [G00226775 image!]).

##### Distribution.

Peru.

##### Notes.

Most of the type specimen deposited at B were destroyed in World War II (Hiepko 1987). The holotype of *Drymaria weberbaueri* at B was probably destroyed; for that reason, Duke (1961) designated a lectotype. This variety is listed as vulnerable (VU) (Cano and Sánchez 2006).

The type of *Drymaria fenzliana* was reportedly collected by Pavon in Mexico, but these are probably duplicates from a collection in Peru; Ruiz and Pavon did not collect in Mexico (Duke 1961).

#### ﻿40c. Drymaria paposana var. serrulata

Taxon classificationPlantaeCaryophyllalesCaryophyllaceae

J.A.Duke, Ann. Missouri Bot. Gard. 48(3): 238. 1961.

A84EC468-A13D-52BD-BC02-288FE8818253

##### Type.

Peru. Arequipa: Caraveli, lomas de Atiquipa (Tamaira), alt. 500–550 m, 27 November 1958, *R. Ferreyra 13490* (holotype: MO [2174593/216510 image!]).

##### Distribution.

Peru.

##### Notes.

This variety is listed as vulnerable (VU) (Cano and Sánchez 2006).

#### ﻿41. Drymaria pattersonii

Taxon classificationPlantaeCaryophyllalesCaryophyllaceae

B.L.Turner, Phytologia 78(3): 201. 1995.

C824CA78-D6C2-5D54-87E1-EF5EB18DB74E

##### Type.

Mexico. Coahuila: ca. 1 km E of the highway at Rancho Santa Lucia along highway 30 between Monclova and Candela, where the road turns SE, 2.3 mi NW of the turnoff to La Carrosa, 23 mi W of the intersection with highway, 26°50'N, 100°47'W, alt. 800–900 m, 18 October 1993, *A. Prather & T. Patterson 1496* (holotype: TEX [00031240 image!].

##### Distribution.

Mexico.

##### Notes.

In the protologue, an isotype was reported from MEXU. However, according to the person in charge of the type collection (Ma. del Rosario García Peña) (pers. comm., 2025), there is no specimen located there.

#### ﻿42. Drymaria perennis

Taxon classificationPlantaeCaryophyllalesCaryophyllaceae

Gilli, Feddes Repert. 92: 677. 1981.

D027F1DB-0727-57E7-82A0-892814C05B0F

##### Type.

Ecuador. Cotopaxi: Zwischen El Corazon und Angamarca, alt. 2600 m, 2 June 1975, *A. Gilli 306* (holotype: W [11292 image!]).

##### Distribution.

Ecuador.

#### ﻿43. Drymaria polycarpoides

Taxon classificationPlantaeCaryophyllalesCaryophyllaceae

A.Gray, Mem. Amer. Acad. Arts 4: 12. 1849.

01D9B472-859F-5A42-929F-7D030E09BF47

≡Mollugophytum polycarpoides (A.Gray) M.E.Jones, Contr. W. Bot. 18: 35. 1933.

##### Type.

Mexico. Durango: Valley of Bolson of Mapimi, s.d., *J. Gregg s.n.* (holotype: GH [00037737 image!]).

##### Distribution.

Mexico.

#### ﻿44. Drymaria praecox

Taxon classificationPlantaeCaryophyllalesCaryophyllaceae

Baehni & J.F.Macbr., Publ. Field Mus. Nat. Hist., Bot. Ser. 13(2/2): 625. 1937.

3E41CC54-E20D-58E1-AFA8-F9E91B944FC4

##### Type.

Peru. Paucartambo: Cuzco, between Pisac and Paucartambo, alt. 4100 m, 23 April 1914, *A. Weberbauer 6916* (holotype: F [548919/F0042714F image!]; isotypes: GH [00037754 image!], MO [2174610/216511 image!], MOL [00229/00000481!], US [1496204/00103358 image!], USM [18409/USM000299!]).

##### Distribution.

Peru.

##### Notes.

The sheet at F was labeled by Duke as an isotype, but it is in fact the holotype, as noted in Duke (1961). This species is currently listed as Endangered (EN) (Cano and Sánchez 2006).

#### ﻿45. Drymaria pratheri

Taxon classificationPlantaeCaryophyllalesCaryophyllaceae

B.L.Turner, Phytologia 78(3): 200. 1995.

917C802C-3224-5845-B125-09C50ECA53FC

##### Type.

Mexico. Nuevo Leon: seven miles NE of Las Estacas (about midway between Monclova and Minas on highway 53) on a private ranch road towards Lechuguillal, 26°25'N, 100°48'W, alt. 650 m, 18 October 1993, *A. Prather, T. Patterson, and A. Hempel 1483* (holotype: TEX [00031239 image!]).

##### Distribution.

Mexico.

#### ﻿46a. Drymaria rotundifolia A.Gray, U. S. Expl. Exped. 123. 1854. var. rotundifolia

Taxon classificationPlantaeCaryophyllalesCaryophyllaceae

3BCED369-148A-55AB-BBE9-28FB405D0371

##### Type.

Peru. [Lima]: Andes, Obrajillo, 1838, *Wilkes Exploring Expedition s.n.* (lectotype, designated by Duke 1961, pg. 239: US [10423/00103360 image!]; isolectotypes: GH [00037756 image!], P [P04936735 image!]).

##### Distribution.

Ecuador, Peru.

##### Notes.

Duke (1961) stated that the specimen in the US was the “HOLOTYPE.” However, the specimens at P, GH, and at US were available to Asa Gray at the time of description and labeled by him as a new species. Thus, we treat Duke’s use of the term holotype as an error to be corrected to lectotype.

#### ﻿46b. Drymaria rotundifolia var. nitida

Taxon classificationPlantaeCaryophyllalesCaryophyllaceae

(Ball) J.A.Duke, Ann. Missouri Bot. Gard. 48(3): 239. 1961.

440629F2-6A90-5BDB-AAA9-3445C73C9464

≡Drymaria nitida Ball, J. Proc. Linn. Soc., Bot. 22: 31. 1887. Type: Peru. [Lima]: Ex saxosis Andium Peruviae juxta pagum Chicla, 12000–13000 ft [alt. 3657.6–3962.4 m], 21–23 April 1882, *J. Ball s.n* (holotype: K [K000471731 !]; isotypes: GH [00037753 image!], LE [LE 00001736 image!], NY [00342518 image!]).

##### Distribution.

Ecuador, Peru.

#### ﻿46c. Drymaria rotundifolia var. mattfeldiana

Taxon classificationPlantaeCaryophyllalesCaryophyllaceae

J.T.Howell, Leaf. W. Bot. 10: 351. 1966.

1F3E4BFA-2AAD-5ABE-B2DB-F94804A511F5

##### Type.

Ecuador. Galapagos Islands: Isabela Island, 4000 ft [alt. 1219.2 m], 26 May 1932, *J. T. Howell 9568* (holotype: CAS [213490/0002332 image!]; isotypes: B [B 10 1226983!], US [2814511/00103361 image!]).

##### Distribution.

Ecuador.

##### Notes.

In the protologue, an isotype was reported from the PRC. However, according to the curator (Patrik Mráz) (pers. comm., 2024), there is no specimen located there.

#### ﻿47. Drymaria stellarioides

Taxon classificationPlantaeCaryophyllalesCaryophyllaceae

Willd. in Roem. & Schult., Syst. Veg. 5: 406. 1819.

4860E0C5-ED2F-57FF-8C96-39BDFC030F29

##### Type.

Ecuador. [Tungurahua]: Crescit prope Hambato, alt. 1380 hex. [alt. 564 m] [Regno Quitensi], [1802], *A. von Humboldt & A. J. A. Bonpland s.n.* (**lectotype, designated here**: P [P00335861!]; isolectotype P [P00335860!]).

##### Distribution.

Ecuador.

##### Notes.

The specimens deposited in P with the label “Herbier Humboldt & Bonpland” come from Bonpland’s private collection. According to Stauffer et al. (2012), these are to be considered the holotypes. In the case of *Drymaria stellarioides*, there are two specimens at P representing original material. However, neither has the label “Herbier Humboldt & Bonpland.” Therefore, we designate the one with the barcode “P00335861” as the lectotype because it is the better specimen and the only one with a flower.

Most of the type specimen deposited at B were destroyed in World War II (Hiepko 1987). Because the holotype at B was probably destroyed, here we designate one of the specimens of P as the lectotype.

For the same reason as with *Drymaria arenarioides*, authorship of *Drymaria stellarioides* should be attributed to Willdenow (see Turland et al. (2018), art. 46.3 example 15).

At F, there is a photo and a fragment probably from the holotype ([980359/0053308F!]). This species is listed as Near Threatened (NT) (León-Yáñez et al. 2011).

#### ﻿48. Drymaria stereophylla

Taxon classificationPlantaeCaryophyllalesCaryophyllaceae

Mattf., Notizbl. Bot. Gart. Berlin-Dahlem 13(118): 436. 1936.

9C347F5F-917F-5242-B95F-266001C97400

≡Drymaria stereophylla Mattf. ex J.F.Macbride, Publ. Field Mus. Nat. Hist., Bot. Ser. 13(2/2): 626. 1937. *nom. illeg.* [non D. stereophylla Mattf. (1936)].
=Drymaria stereophylla var. exstipulata Mattf., Notizbl. Bot. Gart. Berlin-Dahlem 13(118): 437. 1936. Type: Peru. Junin: nordöstlich von Huancayo unterhalb der Hacienda Acopalca, alt. 3600–3700 m, 11 April 1913, *A. Weberbauer 6600* (**lectotype, designated here**: US [1498310/00603668 image!]; isolectotypes: F [627961/F0042716F!], MO [2174586/216509 image!], MOL [00000478 image!], US [1497167/00603667 image!]).

##### Type.

Peru. Junin: La Oroya, 12000 ft [alt. 3657.6 m], 27 May–7 Jun. 1922, *J. F. Macbride & W. Featherstone 962* (**lectotype, designated here**: F [517490/F0042715F image!]; isolectotypes: GH [00037757 image!], NY [00342519 image!], US [1802047/00103363 image!]).

##### Distribution.

Peru.

##### Notes.

Mattfeld described *Drymaria stereophylla* based on a series of specimens collected by A. Raimondi (2502, 2903) and J. F. Macbride and W. Featherstone (563, 962, 1761). However, the protologue does not mention which specimen is the holotype. Furthermore, it is likely that the specimens deposited at B were destroyed in World War II along with most of the type specimens of angiosperms as mentioned by Hiepko (1987) since there are no original specimens there. Thus, the *Macbride & Featherstone* specimen deposited at F [517490/F0042715F!] is designated as the lectotype.

Because the holotype of Drymaria stereophylla var. exstipulata at B was probably destroyed, here we designated one of the specimens at US as the lectotype.

#### ﻿49. Drymaria stipitata

Taxon classificationPlantaeCaryophyllalesCaryophyllaceae

Fosberg, Lloydia 4(4): 281. 1941.

05E49088-152C-5FD8-99A3-71BE8856EEAA

##### Type.

Mexico. Coahuila: mpio. of Sierra Mojada, Sierra Mojada, San Salvador Canyon, 14 September 1939, *C. H. Muller 3301* (holotype: US [2133254/00103364 image!]; isotypes: GH [00037739 image!], LL [00370755 image!], MICH [1115006 image!], MO [3578889/144038 image!], UC [UC 719747 image!]).

##### Distribution.

Mexico.

#### ﻿50. Drymaria subumbellata

Taxon classificationPlantaeCaryophyllalesCaryophyllaceae

I.M.Johnst., J. Arnold Arbor. 31:188. 1950.

3B821508-C5DD-508A-9022-8C43104C263C

##### Type.

Mexico. Coahuila: south end of Cañada Oscuro, near Tanque la Luz, 28 October 1941, *I. M. Johnston 8489* (holotype: GH [00037740 image!]; isotypes: GH [00037741 image!], LL [00370756 image!]).

##### Distribution.

Mexico.

#### ﻿51. Drymaria suffruticosa

Taxon classificationPlantaeCaryophyllalesCaryophyllaceae

A.Gray, Proc. Amer Acad. Arts. 17:328. 1882.

6AD8DE70-AA3A-59D0-93B9-629CC831B1C4

##### Type.

Mexico. Coahuila: San Lorenzo de Laguna to Vicinity, 22 to 27 leagues SW from Parras, (25°26'24"N, 102°10'48"W), 1–10 May 1880, *E. Palmer 74* (holotype: GH [00037742 image!]; isotypes: F [302659/F0053304F image!], K [K000471742 image!], LE [LE 00001731 image!], MO [2174589/144042 image!], NY [00342507 image!, 00342508 image!], US [10422/00103366 image!], VT [UVMVT024425 image!], YU [YU.068841 image!]).

##### Distribution.

Mexico.

#### ﻿52. Drymaria tenuis

Taxon classificationPlantaeCaryophyllalesCaryophyllaceae

S.Watson, Proc. Amer. Acad. Arts 25: 142. 1890.

35BC9EC8-F087-5499-B5E5-CD93871ED32F

≡Drymaria tenuis var. genuina Briq., Annuaire Conserv. Jard. Bot. Genève 13–14: 374. 1911. *nom. invalid.*
=Drymaria filiformis Seaton, Proc. Amer. Acad. Arts 28: 117. 1893. Type: Mexico. Veracruz: Orizaba, 9000 ft [alt. 2743.2 m], 8 August 1891, *H. E. Seaton 267* (holotype: GH [00037725 image!]; isotypes: F [266658/F0053292F image!], K [K000471788!], MO [3578890/144039 image!], NY [00581302 image!], US [1391052/00931428 image!, 10397/00103343 image!]).
=Drymaria tenuis var. jaliscana Briq., Annuaire Conserv. Jard. Bot. Genève 13–14: 374. 1911. Type: Mexico. Jalisco: near Guadalajara, September 1893, *C. G. Pringle 4536* (holotype: G [G00640059 image!]; isotypes: COLO [422760/00363044 image!], E [E00394861 image!], F [264418/F0053305F image!], GH [00037744 image!], KFTA [KFTA0000330 image!], LECB [LECB0000547 image!], MEL [MEL2499838 image!], MICH [1115005 image!], MO [3578893/356353 image!], NY [00342509 image!], PUL [PUL00000233 image!], UC [UC 120343 image!], US [75112/00103367 image!], VT [UVMVT024427 image!]).

##### Type.

Mexico. Jalisco: under ledges of the barranca, near Guadalajara, 28 November 1888, *C. G. Pringle 2120* (holotype: GH [00037743 image!]; isotype: VT [UVMVT024426 image!]).

##### Distribution.

Mexico.

##### Notes.

Duke (1961) mentioned that the holotype of *Drymaria tenuis* is deposited in the US. However, this statement is an error. We have been unable to find a specimen of *Pringle 2120* in the US, and the holotype is in fact the sheet at GH, where Sereno Watson worked at the time the species was described.

#### ﻿53. Drymaria veliziae

Taxon classificationPlantaeCaryophyllalesCaryophyllaceae

Montesinos, PhytoKeys 140: 48. 2020.

2143F10B-CEFC-53CE-8A4F-BBF39D7BEF7F

##### Type.

Peru. Cajamarca: Encañada, Chanta baja, 6°49'56"S, 78°30'20"W, alt. 3295 m, 6 June 2009, *C. Tovar 1058* (holotype: CPUN [22705!]).

##### Distribution.

Peru.

##### Notes.

Currently, this species is listed as endangered (EN) (Montesinos-Tubée et al. 2020).

#### ﻿54a. Drymaria villosa Schltdl. & Cham., Linnaea 5(2): 232. 1830. subsp. villosa

Taxon classificationPlantaeCaryophyllalesCaryophyllaceae

35969A6D-E94B-5038-ACBE-2BE167A97A33

≡Drymaria cordata var. villosa (Schltdl. & Cham.) Rohrb., Fl. Bras. 14(2): 260. 1872.
=Drymaria hirsuta Bartl. in Presl, Reliq. Haenk. 2(1): 8. 1831. Type: Peru: montanis huanoccensibus, 1822, *T. Haenke s.n.* (**lectotype, designated here**: GOET [GOET000583 image!]; isolectotype: PR [24307 image!]).
=Drymaria cubensis Regel, Allg. Gartenzeitung 8(38): 298. 1840. Type: probably Cuba. [Probably cultivated in the Botanical Garden of Berlin from Cuban seed]. (holotype: K [K000471737 image!]).
=Drymaria ciliata C.A.Mey., Index Seminum (St. Petersburg) 9: 71. 1843. Type: This species was described on the basis of cultivated plants known in the horticultural trade.
=Drymaria cordata var. pilosa Schltdl., Linnaea 26: 374. 1853. Type: Mexico. This variety was described in a treatment of miscellaneous Mexican plants.
=Drymaria stylosa Backer, Bull. Jard. Bot. Buitenzorg, sér. 2 12: 15. 1913. Type: Indonesia: many localities in the mountains, s.d., *C. A. Backer s.n.* (without information about type specimens).
=Drymaria tepicana M.E.Jones, Contr. W. Bot. 15: 124. 1929. Type: Mexico: Nayarit: Tepic, 16 February 1927, *M. E. Jones 22847* (holotype: POM [162845/0002390 image!]).
≡Drymaria villosa f. tepicana (M.E.Jones) J.A.Duke, Ann. Missouri Bot. Gard. 48(3): 227. 1961.
=Drymaria barrancae M.E.Jones, Contr. W. Bot. 18: 65. 1933. Type: Mexico. Jalisco: La barranca, Guadalajara, 17 November 1930, *M. E. Jones 27051a* (holotype POM [192857/0002400 image!]; isotype GH 00037718 image!]).

##### Type.

Mexico. Veracruz: Jalapa, without date, *C. J. W. Schiede s.n.* (lectotype, designated by Athira and Maya 2025, pg. 197: LE [LE 00018305 image!]).

##### Distribution.

Subspecies of wide distribution throughout America and Asia.

##### Notes.

*Drymaria villosa* Cham. & Schltdl., originally described in 1830, is equivalent to Drymaria cordata var. villosa (Cham. & Schltdl.) Rohrb. from 1872. In 1961, Duke referred to a specimen at the Komarov Herbarium (LE) as an “isotype,” but according to Article 9.10 of the Shenzhen Code, this should be considered a lectotype. Arya et al. (2024) accepted this correction but incorrectly designated a specimen housed at LECB as the lectotype. However, Chamisso’s original material was transferred to LE (Komarov Institute) in 1840, not to LECB, making their lectotypification invalid (Berger 2018). Athira and Maya (2025) correctly designated specimen LE00018305 from the Komarov Herbarium in St. Petersburg as the lectotype, as it matches Schlechtendal’s original description, ensuring nomenclatural stability. This approach follows Athira and Maya (2025) in recognizing LE00018305 as the lectotype of *D. villosa* Schltdl. & Cham. Finally, although recent plastome sequencing and phylogenetic studies place *D. villosa* within Caryophyllaceae (Chetri et al. 2024), these molecular data do not affect the nomenclatural typification.

#### ﻿54b. Drymaria villosa subsp. palustris

Taxon classificationPlantaeCaryophyllalesCaryophyllaceae

(Schltdl. & Cham.) J.A.Duke, Ann. Missouri Bot. Gard. 48(3): 227. 1961.

FB22C2C3-170F-5081-ADDC-6C0732389DD7

≡Drymaria palustris Schltdl. & Cham., Linnaea 5: 232. 1830. Type: Mexico. Veracruz: In paludosis prope Jalapam, s.d., *C. J. W. Schiede & F. Deppe 504* (lectotype, designated by Duke 1961, pg. 227: LE [LE 00001733 image!]; isolectotype LE [LE 00001732 image!]).
≡Drymaria cordata var. palustris (Schltdl. & Cham.) Rohrb., Fl. Bras. 14(2): 260. 1872.
=Drymaria pauciflora Bartl. in Presl, Reliq. Haenk. 2(1): 8. 1831. Type: Peru: in Peruviae montanis huanoccensibus, 1822, *T. Haenke s.n.* (**lectotype, designated here**: GOET [GOET000582 image!]; isolectotype: PR [24306 image!]).
=Drymaria townsendii B.L.Rob., Bot. Gaz. 30 (1): 58. 1900. Type: Mexico. Chihuahua: Sierra Madre, 5 miles southeast of Colonia Garcia, 7400 ft [alt. 2255.5 m], 5 August 1899, *C. H. T. Townsend & C. M. Barber 231* (holotype: GH [00037745 image!]; isotypes: E [E00326057 image!], F [102975/F0053306F image!], K [K000471738 image!], MO [3578839/144037 image!], NDG [16755 image!], NY [00342510 image!], US [735652/00103368 image!], US [00931459 image!]).
≡Drymaria villosa f. townsendii (B.L.Rob.) J.A.Duke, Ann. Missouri Bot. Gard. 48(3): 228. 1961.
=Drymaria nummularia Briq., Annuaire Conserv. Jard. Bot. Genève 13: 371. 1911. Type: Mexico. [Michoacán]: cordillere d’ Ario, 4000, Jun.–Oct. 1840, *H. G. Galeotti 4416* (holotype: G [G00226781 image!]; isotype: K [3862/K000471748 image!]).
=Drymaria subsessilis M.E.Jones, Contr. W. Bot. 15: 125. 1929. Type: Mexico. Nayarit: in rivulets at Ixtlan, 19 February 1927, *M. E. Jones 22848* (holotype: POM [162843/0002391 image!]; isotype: F [705822/F0053303F image!]).
=Drymaria sphagnophila Baehni & J.F.Macbr., Publ. Field Mus. Nat. Hist., Bot. Ser. 13(2/2): 625. 1937. Type: Peru. Huánuco: Mito, 9000 ft [alt. 2743.2 m], 8–22 July 1922, *J. F. MacBride 1542* (holotype F n. v.; isotype: US [1779692/00103362 image!]).
=Drymaria villosa subsp. palustris var. perennis J.A.Duke, Ann. Missouri Bot. Gard. 48(3): 228. 1961. Type: México. San Luis Potosí: Álvarez, s.d., *E. Palmer 187* (holotype: MO n.v.).

##### Distribution.

Mexico, Guatemala, Honduras, El Salvador, Costa Rica, Panama, Venezuela, Colombia, Ecuador, Peru, Bolivia.

##### Notes.

Most of the type specimen deposited at B were destroyed in World War II (Hiepko 1987), and Duke (1961) mentioned that the holotype of *Drymaria palustris* at B was probably destroyed and designated as the lectotype the specimen cited above. This sheet bears an original label written by Chamisso stating “*Drymaria palustris* n. sp.” (handwriting confirmed by Denise Marx, HAL). However, the number “405” does not correspond to the number given in the protologue, which is “404.” Despite this discrepancy, apparently due to a labeling error, we believe that this specimen is original material and thus agree with Duke’s lectotypification. Although there are two specimens at LE, only one was labeled by Duke ([00001733]). We treat the other ([00001732]) as an isolectotype.

For the same reason as mentioned for *Drymaria apetala*, we designate the specimen in GOET as the lectotype of *Drymaria pauciflora*.

#### ﻿54c. Drymaria villosa subsp. paramorum

Taxon classificationPlantaeCaryophyllalesCaryophyllaceae

(S.F.Blake) J.A.Duke, Ann. Missouri Bot. Gard. 48(3): 230. 1961.

D0C95C4C-8388-5546-AA74-0FCDC89F0EC2

≡Drymaria paramorum S.F.Blake, Contr. U. S. Natl. Herb. 20: 521. 1924. Type: Venezuela. Trujillo: paramo de la cristalina, alt. 2900 m, 20 October 1910, *A. Jahn 111* (holotype: US [602305/00103355 image!]).

##### Distribution.

Colombia, Venezuela.

#### ﻿55. Drymaria viscosa

Taxon classificationPlantaeCaryophyllalesCaryophyllaceae

S.Watson, Proc. Amer. Acad. Arts 22(2): 469. 1887.

5F4ACD1B-4E83-58A6-802D-AF0EEE472049

##### Type.

Mexico. Baja California: Socorro (“Socono”, “Socario”) Northern Lower California, 27 April 1886, *C. R. Orcutt 1330* (holotype: GH [00037746 image!]; isotypes: F [208450/F0053282F image!, 147780/0053283F image!, 219882/0053284F image!], ILL [ILL00007352 image!], MICH [1115004 image!], MIN [410502/1002886 image!], MO [3578903/144036 image!], NY [00342511 image!, 00342512 image!], OS [173559/0000138 image!], UC [UC 8304 image!], US [1391067/00931429 image!, 10383/00103370 image!], VT [UVMVT024429 image!, 024430 image!], YU [YU.001066 image!]).

##### Distribution.

United States, Mexico.

##### Notes.

In Duke (1961), this species is cited as *Drymaria viscosa* S. Watson ex Orcutt. However, Orcutt did not describe the species nor attribute it to Watson, and, although his specimen represents the holotype, it is likely that Watson designated the epithet independently due to the presence of glandular trichomes that give it viscosity (Parfitt and Hodgson 1985).

#### ﻿56. Drymaria xerophylla

Taxon classificationPlantaeCaryophyllalesCaryophyllaceae

A.Gray, Smithsonian Contr. Knowl. 5(6): 18. 1853.

A7D34945-AC43-5BBE-99E0-BFBB0C662BA2

##### Type.

Mexico. Without data, *T. Coulter 722* (holotype: GH [00218128 image!]; isotype: NY [00342513 image!]).

##### Distribution.

Mexico.

### ﻿Excluded and ambiguous names

*Drymaria adiantoides* Muschl, Beibl. Bot. Jahrb. Syst. 111: 5. 1913. According to Duke (1961), this species could be a synonym of *D. villosa*. The protologue mentions that the type is Sodiro no. 127, while Duke mentions that the type collection is Sodiro no. 1291. We have been unable to find original material. Given this confusion and the absence of type specimens, the name is here excluded.

*Drymaria ciliaris* C.A.Mey., Index Seminum (St. Petersburg) 9: 71. 1843. *nom. invalid.* This name was never validly published and was merely mentioned as a name that could be found in the horticultural trade. It probably corresponds to *Drymaria villosa*.

*Drymaria filiformis* Benth., Fl. Austral. 1:162. 1863. This is *Stellaria filiformis* (Benth.) Mattf., an Asian species.

*Drymaria grandiflora* Schltdl., Linnaea 12: 205. 1838. *nom. illeg.* [non *D. grandiflora* Bartl. (1831)]. *Drymaria megalantha* Steud., Nomencl. Bot., ed. 2, 1: 532. 1840. No collection information was provided in the protologue, and we have been unable to find type specimens. However, according to the description, we concur with Duke (1961) that is possible *Stellaria*.

*Drymaria idiopoda* S.F.Blake, Contr. U. S. Nat. Herb. 24(1): 4. 1922. Based on the observation of the type specimens, this species corresponds to *Stellaria ovata* Willd. ex D.F.K.Schltdl.

*Drymaria mairei* H.Lév. This species corresponds to *Odontostemma mairei* (H.Lév.) Rabeler & W.L.Wagner.

*Drymaria oxalidea* Pax, Bot. Jahrb. Syst. 18: 31. 1893. Due to the lack of information about this species, the absence of type specimens that were probably destroyed in herbarium B during World War II (Hiepko 1987), and the lack of a detailed description presented in the protologue, we are unable to determine the identity of this name.

*Drymaria palmeri* Hemsl., Diagn. Pl. Nov. Mexic. 2: 22. 1879. Based on the observation of the type specimens, this species corresponds to *Stellaria ovata*.

*Drymaria rotundifolia* (Poir.) Har., J. Bot. (Morot) 14: 150. 1900. We concur with Duke (1961) that this is *Stellaria rotundifolia* Poir.

## Supplementary Material

XML Treatment for Drymaria

XML Treatment for Drymaria anilii

XML Treatment for Drymaria anomala

XML Treatment for Drymaria apetala

XML Treatment for Drymaria arenarioides Willd. in Roem. & Schult., Syst. Veg. 5: 406. 1819. subsp. arenarioides

XML Treatment for Drymaria arenarioides subsp. peninsularis

XML Treatment for Drymaria auriculipetala

XML Treatment for Drymaria axillaris

XML Treatment for Drymaria barkleyi

XML Treatment for Drymaria coahuilana

XML Treatment for Drymaria conzattii

XML Treatment for Drymaria cordata (L.) Willd. in Roem. & Schult., Syst. Veg. 5: 406. 1819. subsp. cordata

XML Treatment for Drymaria cordata subsp. diandra

XML Treatment for Drymaria cubana

XML Treatment for Drymaria debilis

XML Treatment for Drymaria divaricata

XML Treatment for Drymaria divaricata var. stricta

XML Treatment for Drymaria divaricata var. viscidula

XML Treatment for Drymaria divaricata var. divergens

XML Treatment for Drymaria divaricata var. reflexiflora

XML Treatment for Drymaria effusa A.Gray, Smithsonian Contr. Knowl. 5(6): 19. 1853. var. effusa

XML Treatment for Drymaria effusa var. confusa

XML Treatment for Drymaria effusa var. depressa

XML Treatment for Drymaria elata

XML Treatment for Drymaria engleriana (Muschl.) Baehni & J.F.Macbr., Publ. Field Mus. Nat. Hist., Bot. Ser. 13(2/2): 621. 1937. var. engleriana

XML Treatment for Drymaria engleriana var. devia

XML Treatment for Drymaria excisa

XML Treatment for Drymaria fasciculata

XML Treatment for Drymaria firmula

XML Treatment for Drymaria frutescens

XML Treatment for Drymaria glaberrima

XML Treatment for Drymaria glandulosa Phil., Fl. Atacam. 10. 1860. var. glandulosa

XML Treatment for Drymaria glandulosa var. galeottiana

XML Treatment for Drymaria gracilis Phil., Fl. Atacam. 10. 1860. subsp. gracilis

XML Treatment for Drymaria gracilis subsp. carinata

XML Treatment for Drymaria grandiflora

XML Treatment for Drymaria holosteoides Benth., Bot. Voy. Sulphur: 16. 1844. var. holosteoides

XML Treatment for Drymaria holosteoides var. crassifolia

XML Treatment for Drymaria hypericifolia

XML Treatment for Drymaria jenniferae

XML Treatment for Drymaria ladewii

XML Treatment for Drymaria laxiflora

XML Treatment for Drymaria leptophylla (Cham. & Schltdl.) Fenzl ex Rohrb., Linnaea 37(2): 195. 1871–1873 [1872]. var. leptophylla

XML Treatment for Drymaria leptophylla var. cognata

XML Treatment for Drymaria leptophylla var. nodosa

XML Treatment for Drymaria longepedunculata

XML Treatment for Drymaria lyropetala

XML Treatment for Drymaria malachioides

XML Treatment for Drymaria molluginea

XML Treatment for Drymaria monticola

XML Treatment for Drymaria multiflora

XML Treatment for Drymaria ortegioides

XML Treatment for Drymaria ovata

XML Treatment for Drymaria pachyphylla

XML Treatment for Drymaria paposana Phil., Fl. Atacam. 10. 1860. var. paposana

XML Treatment for Drymaria paposana var. weberbaueri

XML Treatment for Drymaria paposana var. serrulata

XML Treatment for Drymaria pattersonii

XML Treatment for Drymaria perennis

XML Treatment for Drymaria polycarpoides

XML Treatment for Drymaria praecox

XML Treatment for Drymaria pratheri

XML Treatment for Drymaria rotundifolia A.Gray, U. S. Expl. Exped. 123. 1854. var. rotundifolia

XML Treatment for Drymaria rotundifolia var. nitida

XML Treatment for Drymaria rotundifolia var. mattfeldiana

XML Treatment for Drymaria stellarioides

XML Treatment for Drymaria stereophylla

XML Treatment for Drymaria stipitata

XML Treatment for Drymaria subumbellata

XML Treatment for Drymaria suffruticosa

XML Treatment for Drymaria tenuis

XML Treatment for Drymaria veliziae

XML Treatment for Drymaria villosa Schltdl. & Cham., Linnaea 5(2): 232. 1830. subsp. villosa

XML Treatment for Drymaria villosa subsp. palustris

XML Treatment for Drymaria villosa subsp. paramorum

XML Treatment for Drymaria viscosa

XML Treatment for Drymaria xerophylla

## ﻿Additional information

### Use of AI

No use of AI was reported.

### Author ORCIDs

Dagoberto Valentín-Martínez https://orcid.org/0000-0002-7355-5627

Victor W. Steinmann https://orcid.org/0000-0003-0103-9575

Hilda Flores-Olvera https://orcid.org/0000-0002-3262-9570

Thomas Borsch https://orcid.org/0000-0002-5724-2786

Daniel B. Montesinos-Tubée https://orcid.org/0000-0002-4439-5089

### Data availability

All of the data that support the findings of this study are available in the main text.

## References

[B1] Arya S Singh H Walsan-Vishnu K Iamonico D (2024) Study on the Genus *Drymaria* (Caryophyllaceae)—A New Species from North-East India. Plants 13(23): 3378. 10.3390/plants13233378 39683172 PMC11644691

[B2] Athira S Maya CN (2025) Lectotypification of *Arenaria neelgherrensis* and *Drymaria villosa* (Caryophyllaceae). Annales Botanici Fennici 62: 195–198. 10.5735/085.062.0131

[B3] Bartling FG (1831) Alsineae. In: Presl KB (Ed.) Reliquiae Haenkeanae; seu, Descriptiones et icones plantarum: quas in America meridionali et boreali, in insulis Philippinis et Marianis collegit.Thaddaeus Haenke, Pragae, 1–152.

[B4] Berger A (2018) Synopsis and typification of Mexican and Central American Palicourea (Rubiaceae: Palicoureeae), part I: the entomophilous species. Annalen des Naturhistorischen Museums in Wien. Serie B für Botanik und Zoologie 120: 59–140.

[B5] Borsch T Hernández-Ledesma P Berendsohn WG Flores-Olvera H Ochoterena H Zuloaga FO von Mering S Kilian N (2015) An integrative and dynamic approach for monographing species-rich plant groups – building the global synthesis of the angiosperm order Caryophyllales. Perspectives in Plant Ecology, Evolution and Systematics 17(4): 284–300. 10.1016/j.ppees.2015.05.003

[B6] Britton NL Millspaugh CF (1920) The Bahama Flora. New York: The Authors, U.S. 1–695. 10.5962/bhl.title.54084

[B7] Brockington SF Alexandre R Ramdial J Moore MJ Crawley S Dhingra A Hilu K Soltis DE Soltis PS (2009) Phylogeny of the Caryophyllales Sensu Lato: Revisiting hypotheses on pollination biology and perianth differentiation in the core Caryophyllales. International Journal of Plant Sciences 170(5): 627–643. 10.1086/597785

[B8] Cafferty S Jarvis CE (2004) Typification of Linnean plant names in *Caryophyllaceae*. Taxon 53(4): 1049–1054. 10.2307/4135573

[B9] Calderón de Rzedowski G Rzedowski J (2005) Flora fanerogámica del Valle de México. Instituto de Ecología, A. C. / Comisión Nacional para el Conocimiento y Uso de la Biodiversidad, 1–1406.

[B10] Cano A Sánchez I (2006) Caryophyllaceae endémicas del Perú. Revista Peruana de Biología 13(2): 246–252. 10.15381/rpb.v13i2.1835

[B11] Castro-Mendoza I Fonseca RM (2012) Caryophyllaceae. Flora de Guerrero 48. Universidad Nacional Autónoma de México, Facultad de Ciencias, 1–62.

[B12] Chetri BK Sonu SS Shelke RG Mitra S Rangan L (2024) De novo sequencing of *Drymaria villosa* and comparative analysis of plastome in Caryophyllaceae across 23 Species. Molecular Biotechnology, 1–9. 10.1007/s12033-024-01317-0 39516423

[B13] Cuénoud P Savolainen V Chatrou LW Powell M Grayer RJ Chase MW (2002) Molecular Phylogenetics of Caryophyllales Based on Nuclear 18S rDNA and Plastid rbcL, atpB, and matK DNA Sequences. American Journal of Botany 89(1): 132–144. 10.3732/ajb.89.1.132 21669721

[B14] Didrichsen DF (1858) Index seminum in h. acad. Huaniensi. Linnaea 29: 737–738. https://www.biodiversitylibrary.org/page/116358#page/1/mode/1up

[B15] Downie SR Katz-Downie DS Cho K-J (1997) Relationships in the Caryophyllales as Suggested by Phylogenetic Analyses of Partial Chloroplast DNA ORF2280 Homolog Sequences. American Journal of Botany 84(2): 253–273. 10.2307/2446087 21712205

[B16] Duke JA (1961) Preliminary Revision of the Genus *Drymaria.* Annals of the Missouri Botanical Garden 48(3): 173–268. 10.2307/2394953

[B17] Escamilla M Sosa V (2000) Características de las semillas en las series del género *Drymaria* (Caryophyllaceae). Kurtziana 28: 259–274.

[B18] Fior S Karis PO Casazza G Minuto L Sala F (2006) Molecular phylogeny of the Caryophyllaceae (Caryophyllales) inferred from chloroplast matK and nuclear rDNA ITS sequences. American Journal of Botany 93(3): 399–411. 10.3732/ajb.93.3.399 21646200

[B19] Flores-Olvera H Ochoterena H Arias S Berendsohn WG Borsch T von Mering S Zuloaga FO (2018) The Caryophyllales 2018 Conference in Mexico & Advances of the Global Caryophyllales Initiative. Taxon 67(6): 1250–1255. 10.12705/676.44

[B20] Fosberg FR (1945) *Drymaria* and Mollugophytum. In: Morton CV Mexican phanerogams described by M. E. Jones.Contributions from the United States National Herbarium 29: 95–96. https://www.biodiversitylibrary.org/page/397425#page/1/mode/1up

[B21] GBIF (2025) GBIF Occurrence Database. GBIF | Global Biodiversity Information Facility. Copenhagen Denmark. http://www.gbif.org [accessed 10.02.2025]

[B22] Gray A (1854) United States Exploring Expedition. During the years 1838, 1839, 1840, 1841, 1842. Under the Command of Charles Wilkes, U.S.N. vol. XV. Botany. Phanerogamia by Asa Gray with a Folia Atlas of 100 Plates. Part 1, 1–128.

[B23] Greenberg AK Donoghue MJ (2011) Molecular systematics and character evolution in Caryophyllaceae. Taxon 60(6): 1637–1652. 10.1002/tax.606009

[B24] Harbaugh DT Nepokroeff M Rabeler RK McNeill J Zimmer EA Wagner WL (2010) A new linage-based tribal classification of the family Caryophyllaceae. International Journal of Plant Sciences 171(2): 185–198. 10.1086/648993

[B25] Hartman RL (2005) *Drymaria*. In: Flora of North America Editorial Committee (Ed.). Flora of North America north of Mexico, Oxford University Press, New York, 9–15. Harvard University and Herbaria and Libraries. https://kiki.huh.harvard.edu/databases/image_search.php [accessed 28.12.2024]

[B26] Hernández-Ledesma P Berendsohn WG Borsch T Von Mering S Akhani H Arias S Castañeda-Noa I Eggli U Eriksson R Flores-Olvera H Fuentes-Bazán S Kadereit G Klak C Korotkova N Nyffeler R Ocampo G Ochoterena H Oxelman B Rabeler RK Sanchez A Schlumpberger BO Uotila P (2015) A taxonomic backbone for the global synthesis of species diversity in the angiosperm order Caryophyllales. Willdenowia 45(3): 281–383. 10.3372/wi.45.45301

[B27] Hiepko P (1987) The Collections of the Botanical Museum Berlin-Dahlem (B) and their history. Englera 7: 219–252.

[B28] ITHAKA (2024) JSTOR Global Plants. ITHAKA, New York. http://plants.jstor.org [accessed 28.12.2024]

[B29] Johnston IM (1940) New phanerogams from Mexico, II. Journal of the Arnold Arboretum 21(1): 67–75. 10.5962/p.37226

[B30] Johnston IM (1950) Noteworthy species from Mexico and adjacent United States, III. Journal of the Arnold Arboretum 31(2): 188–195. 10.5962/p.333924

[B31] Jones ME (1933) Contributions to Western Botany 18. Biogeographical sketch of Prof. Jones by Mabel Jones Broaddus, 1–157. https://www.biodiversitylibrary.org/page/10608223#page/189/mode/1up

[B32] Kunth CS (1823) Caryophylleae. In: Bonpland A, Humboldt A. Kunth CS (Eds) Nova Genera et Species Plantarum 6. Paris, 19–37. https://www.biodiversitylibrary.org/page/7046#page/42/mode/1up

[B33] León-Yáñez S Valencia R Pitman N Endara L Ulloa Ulloa C Navarrete H (2011) Libro rojo de las plantas endémicas del Ecuador. Pontificia Universidad Católica del Ecuador, Quito, 1–440.

[B34] Linnaeus C (1753) Species Plantarum (Vol. 1). Impensis Laurentii Salvii, 1–560.

[B35] Liogier HA (1960) Novedades en la Flora cubana. Candollea 17: 99–111.

[B36] Liogier HA (1962) Novelties in the Cuban flora. XIV. Phytologia 8(7): 369–370.

[B37] Macbride JF (1937) Caryophyllaceae. Flora of Peru. Publication. Field Museum of Natural History. Botanical Series 13, part II (2): 578–638. 10.5962/bhl.title.2277

[B38] Machuca-Machuca K (2024) Caryophyllaceae. Flora del Valle de Tehuacán-Cuicatlán 192, Instituto de Biología, Universidad Nacional Autónoma de México, 1–40. 10.22201/ib.9786073096416e.2024

[B39] Mattfeld J (1936) Einige neue *Drymaria*-Arten aus Peru. Notizblatt des Botanischen Gartens und Museums zu Berlin-Dahlem 13(118): 436–444. 10.2307/3995022

[B40] Mizushima S (1957) A revision of *Drymaria cordata* Willd. Shokubutsu Kenkyu Zasshi 32(3): 69–81. 10.51033/jjapbot.32_3_4108

[B41] Montesinos-Tubée DB Tovar C Iberico-Vela G Montoya-Quino J Sanchez-Vega I (2020) *Drymaria veliziae* (Caryophyllaceae), a new species from the Andes of Cajamarca (North Peru). PhytoKeys 140: 47–56. 10.3897/phytokeys.140.47738 32148431 PMC7052040

[B42] Muñoz-Schick M Moreira-Muñoz A Moreira-Espinoza S (2012) Origen del nombre de los géneros de plantas vasculares nativas de Chile y su representatividad en Chile y el mundo. Gayana. Botánica 69(2): 309–359. 10.4067/S0717-66432012000200011

[B43] Parfitt BD Hodgson W (1985) *Drymaria viscosa* (Caryophyllaceae): Correct author citation and range extension to the United States. SIDA, Contributions to Botany 11(1): 96–98. https://ia802807.us.archive.org/1/items/biostor-158150/biostor-158150.pdf

[B44] Pérez-Calix E Grajales-Tam KM (2013) Caryophyllaceae. Flora del Bajío y de Regiones Adyacentes 180, Instituto de Ecología A.C., Centro Regional del Bajío, 1–122.

[B45] Rodríguez-Jiménez C (2013) Caryophyllaceae. In: Flora Mesoamericana 2(1): 1–68.

[B46] Roemer JJ Schultes JA (1819) Systema Vegetabilium. Sumtibus J.G. Cottae, 1–632. https://www.biodiversitylibrary.org/page/715118#page/1/mode/1up

[B47] Sandoval-Ortega MH Siqueiros-Delgado ME Cerros-Tlatilpa R Ocampo G (2019) La familia Caryophyllaceae en el estado de Aguascalientes, México. Acta Botánica Mexicana 126(126): e1455. 10.21829/abm126.2019.1455

[B48] Seringe NC (1824) Caryophylleae. In: Candolle AP de (Ed.) Prodromus Systematis Naturalis Regni Vegetabilis: sive enumeratio contracta ordinum generum specierumque plantarum huc usque cognitarium, juxta methodi naturalis, normas digesta.Truettel et Würtz, Paris, 351–422. https://www.biodiversitylibrary.org/page/154356#page/5/mode/1up

[B49] Stafleu FA Cowan RS (1976) Taxonomic literature: a selective guide to botanical publications and collections with dates, commentaries and types. Vol. 1: A-G. Regnum Vegetabile 94: 1–1136. 10.5962/bhl.title.48631 [Bohn, Scheltema & Holkema.]

[B50] Stafleu FA Cowan RS (1979) Taxonomic literature: a selective guide to botanical publications and collections with dates, commentaries and types. Vol. 2: H-Le. Regnum Vegetabile 98: 1–991. 10.5962/t.206091 [Bohn, Scheltema & Holkema.]

[B51] Stafleu FA Cowan RS (1981) Taxonomic literature: a selective guide to botanical publications and collections with dates, commentaries and types. Vol. 3: Lh-O. Regnum Vegetabile 105: 1–980. 10.5962/t.207599 [Bohn, Scheltema & Holkema.]

[B52] Stafleu FA Cowan RS (1983) Taxonomic literature: a selective guide to botanical publications and collections with dates, commentaries and types. Vol. 4: P-Sak. Regnum Vegetabile 110: 1–1214. 10.5962/t.206493 [Bohn, Scheltema & Holkema.]

[B53] Stafleu FA Cowan RS (1986) Taxonomic literature: a selective guide to botanical publications and collections with dates, commentaries and types. Vol. 5: Sal-Ste. Regnum Vegetabile 112: 1–1066. [Bohn, Scheltema & Holkema.] 10.5962/t.206495

[B54] Stafleu FA Cowan RS (1988) Taxonomic literature: a selective guide to botanical publications and collections with dates, commentaries and types. Vol. 6: Sti-Vuy. Regnum Vegetabile 115: 1–926. [Bohn, Scheltema & Holkema.] 10.5962/t.206496

[B55] Standley PC Steyermark JA (1946) Caryophyllaceae. Flora of Guatemala. Fieldiana. Botany 24(4): 217–239. 10.5962/bhl.title.2233

[B56] Stauffer FW Stauffer J Dorr LJ (2012) Bonpland and Humboldt specimens, field notes, and herbaria; new insights from a study of the monocotyledons collected in Venezuela. Candollea 67(1): 75–130. 10.15553/c2012v671a10

[B57] Swartz O (1788) Nova genera et species plantarum seu Podromus. M. Swederi, 1–152. https://www.biodiversitylibrary.org/page/376746#page/161/mode/1up

[B58] Thiers B (2025) Index Herbariorum: A global directory of public herbaria and associated staff. New York Botanical Garden’s Virtual Herbarium, New York. http://sweetgum.nybg.org/science/ih [accessed 20.02.2025]

[B59] Turland NJ Wiersema JH Barrie FR Greuter W Hawksworth DL Herendeen PS Knapp S Kusber W-H Li D-Z Marhold K May TW McNeill J Monro AM Prado J Price MJ Smith GF (2018) International Code of Nomenclature for Algae, Fungi and Plants (Shenzhen Code) adopted by the Nineteenth International Botanical Congress Shenzhen, China, July 2017. Regnum Vegetabile 159. Koeltz Botanical Books, Glashütten. 10.12705/Code.2018

[B60] Turner BL (1995) Two new species of *Drymaria* (Caryophyllaceae) from gypseous soils in northern Nuevo Leon, Mexico. Phylologia 78(3): 199–203. https://biostor.org/reference/63468

[B61] Urban I (1930) Plantae Haitienses et Domingenses. Arkiv för Botanik 23A(5): 1–107.

[B62] Villarreal JA Estrada AE (2008) A new species of *Drymaria* (Caryophyllaceae) from northeastern Mexico. Brittonia 60(4): 329–331. 10.1007/s12228-008-9028-x

[B63] Wiggins IL (1944) The genus *Drymaria* in and adjacent to the Sonoran Desert. Proceedings of the California Academy of Sciences 25(6): 189–214. https://biostor.org/reference/78130

[B64] Zanotti CA Moroni P Acosta JM (2022) Estudios morfológicos y moleculares respaldan la presencia del género *Microphytes* en la Argentina y su posición filogenética en la tribu Polycarpeae (Caryophyllaceae). Darwiniana, n. s. 10(2): 388–403. 10.14522/darwiniana.2022.102.1079

